# Cellular microenvironment: a key for tuning mesenchymal stem cell senescence

**DOI:** 10.3389/fcell.2023.1323678

**Published:** 2023-12-04

**Authors:** Wenyang Sun, Jiacheng Lv, Shu Guo, Mengzhu Lv

**Affiliations:** Department of Plastic Surgery, The First Hospital of China Medical University, Shenyang, Liaoning, China

**Keywords:** mesenchymal stem cells, tissue engineering, SASPs, cellular senescence, cellular microenvironment

## Abstract

Mesenchymal stem cells (MSCs) possess the ability to self-renew and differentiate into multiple cell types, making them highly suitable for use as seed cells in tissue engineering. These can be derived from various sources and have been found to play crucial roles in several physiological processes, such as tissue repair, immune regulation, and intercellular communication. However, the limited capacity for cell proliferation and the secretion of senescence-associated secreted phenotypes (SASPs) pose challenges for the clinical application of MSCs. In this review, we provide a comprehensive summary of the senescence characteristics of MSCs and examine the different features of cellular microenvironments studied thus far. Additionally, we discuss the mechanisms by which cellular microenvironments regulate the senescence process of MSCs, offering insights into preserving their functionality and enhancing their effectiveness.

## 1 Introduction

Over the past decade, there has been significant progress in the field of stem cell-based regenerative therapeutics and *ex vivo* disease models. This progress has been achieved by leveraging the pluripotent and immunological features of stem cells. Mesenchymal stem cells (MSCs), which are adult non-hematopoietic mesodermal stem cells, were first isolated from bone marrow in 1968. (Friedenstein et al., 1968). MSCs offer numerous advantages over other types of stem cells in terms of therapy and applications. These versatile cells can be derived from various sources such as bone marrow, umbilical cord, placenta, fat, cartilage, skin, lungs, and dental pulp. (Pittenger et al., 2019; Liu Yajun et al., 2022). Previous research has shown that genetic alteration of MSCs and gene delivery are both feasible and practical (Damasceno et al., 2020; Zhou et al., 2023). The immunological flexibility of MSCs makes them highly effective in regulating and improving the inflammatory microenvironment. (Zhuang et al., 2022). To date, treatments using MSCs and their extracellular vesicles (EVs) have demonstrated beneficial effects in a variety of diseases, such as osteoarthritis (OA) (Greif et al., 2020; Kim et al., 2020; Rizzo et al., 2023), diabetic mellitus (DM) (Sun F. et al., 2022; Yang et al., 2022), Crohn’s disease (CD) (Garcia-Olmo et al., 2022; Huang et al., 2022), systemic lupus erythematosus (SLE) (Zhang Mingchao et al., 2022), myocardial infarction (MI) (Czosseck et al., 2022), acute respiratory distress disorder (ARD) (Jackson et al., 2016; Qiao et al., 2021) and graft-versus-host disease (GVHD) (Harrell et al., 2022). In 2006, the International Society for Cellular Therapy (ISCT) proposed minimal criteria to define MSCs. These criteria include: i. MSCs should adhere to plastic under standard culture conditions; ii. MSCs should express CD105, CD73, and CD90, while not expressing CD45, CD34, CD14 or CD11b, CD79 or CD19, and HLA-DR surface markers; and iii. MSCs should demonstrate the ability to differentiate into three different lineages: osteogenic, lipogenic, and chondrogenic. (Dominici et al., 2006; Mareschi et al., 2006).

However, as MSCs undergo senescence, they also undergo a transition from an anti-inflammatory to a pro-inflammatory factor phenotype. This shift can reduce the immunomodulatory potential of MSCs and significantly limit the effectiveness of stem cell therapy. Moreover, senescent MSCs alter the cellular microenvironment surrounding them through the secretion of senescence-associated secreted phenotypes (SASPs), the generation of reactive oxygen species (ROS), and the remodeling of the extracellular matrix (Liu J. et al., 2022; Siraj et al., 2023). These changes induce senescence in non-senescent MSCs. As a result, stringent criteria may be necessary for creating high-quality MSCs for therapeutic applications and obtaining large quantities of high-purity MSCs from patients can be challenging. (Zhang Y. et al., 2021).

Efforts to enhance the efficiency of cell therapy must address the negative effects of senescence in MSCs. Multiple factors contribute to the senescence of MSCs, including cell-intrinsic regulatory mechanisms related to DNA damage, telomere shortening, and epigenetic modifications. Additionally, the cellular microenvironment in which MSCs reside plays a significant role in influencing their senescence-related behaviors. The cellular microenvironment refers to the local region consisting of neighboring cells and non-cellular components. It provides structural support and signaling that are crucial for maintaining the homeostasis and functionality of MSCs(Choi et al., 2014; Papait et al., 2020; Liu J. et al., 2022; Pei et al., 2023). Creating an appropriate cellular microenvironment is also essential for advancements in tissue engineering and regenerative medicine. The state and function of MSCs can be influenced by changes in the cellular microenvironment during various physiological and pathological conditions (Papait et al., 2020; Liu J. et al., 2022; Tan et al., 2022). The current body of literature does not provide a comprehensive overview of the impact of the cellular microenvironment on senescence in MSCs. As a result, the objective of this paper is to compile and summarize the effects and mechanisms of different cellular microenvironments on the senescence and behavior of MSCs. Additionally, this paper will explore potential avenues for future research in this field.

## 2 Characteristics of MSCs senescence

Cellular senescence is a physiological state that manifests as a stable cell cycle stagnation (Herranz and Gil, 2018). The discovery can be traced back to the 1960s, when Haflick and Moorhead experimentally cultured and observed human fibroblasts' inability to divide indefinitely. This phenomenon is known as the 'Hayflick limit'. (Hayflick and Moorhead, 1961). Senescent cells are characterized by heightened intracellular expression of senescence-related genes, such as p16 and p53 (Hernandez-Segura et al., 2018), as well as senescence-associated β-galactosidase (SA-β-gal). Additionally, they further induce the surrounding cells and microenvironment into senescence through paracrine effects. (Di Mitri and Alimonti, 2016).

Previous research has demonstrated that the presence and elimination of senescent cells have a beneficial effect on maintaining the microenvironment and organ function in the human body over a short period of time. However, the long-term accumulation of senescent cells can have the opposite effect and contribute to the development of age-related diseases (ARDs) (Yin Yujia et al., 2021). Cellular senescence is also a contributing factor to individual aging, a gradual decline in physiological function. Previous studies have demonstrated the effectiveness of Senolytics in eliminating senescent cells and addressing ARDs such as cardiovascular diseases, metabolic diseases, and frailty (Zhu et al., 2015; Chaib et al., 2022). For instance, the application of ABT-263 as a pretreatment for synovial MSCs has proven to be successful in eliminating senescent cells and enhancing the outcomes of patients with OA (Miura et al., 2022). Lopez-Otin et al. (Lopez-Otin et al., 2013) identified nine hallmarks of aging, including DNA damage, telomere attrition, epigenetic modification, loss of proteostasis, mitochondrial failure, cellular senescence, nutrition sensing, intracellular communication, and stem cell exhaustion. Cellular senescence and stem cell exhaustion are the main mechanisms of aging. In this section, we will focus on the characteristics of MSCs' senescence.

### 2.1 Cell cycle arrest

Unlike cells in a quiescent state, senescent cells are metabolically active but arrested in the G1/S phase of the cell cycle. This arrest is primarily caused by activation of the p53/p21CIP1 signaling pathway or the p16^INK4A/Rb^ oncogenic pathway (Kamal et al., 2020; Engeland, 2022). p53, a tumor suppressor gene, promotes genomic stability by inducing cell cycle arrest and apoptosis (Chen, 2016). It also plays a significant role in cellular senescence and aging (Hafner et al., 2019). In p53-induced senescent MSCs, the expression of p53-regulated downstream miRNAs (such as miR-34a/b/c, miR-29, miR-145, and miR-192) increases, with miR-34a showing the strongest association with p53. Overexpression of miR-34a in MSCs inhibits osteogenic differentiation and accelerates senescence (Xia et al., 2021). Interestingly, the expression of p16^INK4A^ is elevated in P3 MSCs overexpressing miR-34a, while the expression of p21WAF1/CIP remains largely unchanged, suggesting different mechanisms for replicative senescence (Pi et al., 2021). Apart from p53-dependent miRNAs, other miRNAs also play distinct roles in MSC senescence. For instance, miR-200c-3p inhibits the p53/p21 axis and enhances the transcription of stemness-related genes (Nanog, Oct4, and Sox2) (Anastasiadou et al., 2021).

### 2.2 Morphological and biological changes

There is a strong correlation between the morphology and function of MSCs. Previous studies have shown that larger MSCs have similar ATP levels and SA-β-gal activity as those obtained from older individuals (Liu J. et al., 2020). On the other hand, smaller MSCs resemble those obtained from younger individuals, suggesting that cell size could be used as a potential indicator to evaluate the level of senescence in MSCs (Block et al., 2017; Zhai et al., 2021). Senescent MSCs undergo a transformation from their typical spindle shape to an enlarged, irregular, flattened form (Li et al., 2017). As the number of passages increases, the shape of human adipose-derived stem cells (ADSCs) changes, with the emergence of pseudopod structures in the 10th generation and a 'fried egg' morphology in the 15th generation. Additionally, prolonged culture significantly reduces cell density in the same magnification field, while the cell diameter gradually increases (Truong et al., 2019).

### 2.3 Senescence-associated-β-galactosidase

Senescence-associated-β-galactosidase (SA-β-gal) was initially discovered by Dimri et al., in 1995 (Dimri et al., 1995). Since then, it has emerged as the most extensively utilized biomarker for identifying senescent cells, both *in vitro* and *in vivo*. SA-β-gal activity is highly correlated with senescent cells and is not detectable in quiescent or differentiated cells. The level of SA-β-gal is associated with the amount of lysosomes inside cells (Lozono-Torres et al., 2019), and the increase in size and volume of lysosomes is primarily due to the presence of lipofuscin, a marker for senescent cells (Vida et al., 2017). Aspirin (2-Acetoxybenzoic acid) therapy was found to effectively reduce the number of SA-β-gal-positive cells in replicative senescent bone marrow stem cells (BMSCs). Additionally, there was a significant decrease in the expressions of p16, p53, and p21, and the blue staining of BMSCs nuclei was also reduced (Liu X. P. et al., 2022).

### 2.4 Colony-forming ability

The ability of a single cell to proliferate *ex vivo* for more than six generations and its progeny to form a population of cells is commonly referred to as a 'colony' or 'clone.' The colony formation rate serves as an indicator of the cell’s ability to survive independently. Colony-forming ability is considered a significant characteristic of cell stemness, and MSCs tend to lose their colony-forming ability as their proliferation decreases and they enter senescence (Ridzuan et al., 2016). In a study conducted by Kapetanou et al., proteasomal changes associated with senescence were observed in late-passaged (p40) human mesenchymal stem cells (hMSCs). The researchers found a decrease in the expression of the β5 subunit, which was closely linked to the poor proliferative potential of hMSCs. However, when β5 was overexpressed in Wharton’s jelly-derived mesenchymal stem cells (WJ-MSCs), the senescence-associated proteasomal alterations were rescued. This overexpression of β5 not only enhanced the stemness and proliferative capacity of late-passaged MSCs, but it also resulted in a decrease in proteasomal oxidative protein modifications and intracellular ROS levels (Kapetanou Marianna et al., 2017). Furthermore, it has been demonstrated that the ability of MSCs to form colonies is also influenced by telomere length (Guerrero et al., 2021) and the consistent expression of proto-oncogenes, such as B cell lymphoma 3 (Bcl-3) (Wang Fuxiao et al., 2022).

### 2.5 Differentiation bias

The ability of MSCs to differentiate into three lineages is a distinguishing characteristic, and an imbalance in the differentiation into osteogenic and lipogenic lineages is a problem associated with MSC senescence. MSCs in the bone marrow (BM) microenvironment show reduced proliferative capacity and increased SA-β-gal activity, which are linked to age-related osteoporosis and fractures. Additionally, there is a decrease in osteogenic differentiation and an increased tendency towards lipogenic differentiation (Yi et al., 2021). Bcl-3, an inhibitor of NF-κB that plays a role in maintaining Wnt/β-catenin signaling and promoting osteogenic differentiation while inhibiting lipogenic differentiation in BMSCs, is considered a crucial target for treating age-related osteoporosis (Chen Xi et al., 2020; Jing et al., 2022).

In recent years, researchers have postulated that several miRNAs affect biological characteristics in MSCs through various signaling pathways (Yang et al., 2021). One such miRNA, miR-204, has been found to be significantly increased in senescent cells and has regulatory effects on SASPs factors such as IL-6 and MMP-3 (Kang et al., 2019). Additionally, it has been observed to inhibit the osteogenic differentiation of BMSCs by regulating RUNX2 (Huang et al., 2010). Another important discovery is the role of the long noncoding RNA zinc finger antisense 1 (ZFAS1) in governing the osteogenic development of BMSCs through the ZFAS1-miR-499-EPHA5 axis. This finding suggests that targeting ZFAS1 could be crucial in the treatment of osteoporosis in the elderly, as BMSCs with ZFAS1 knockdown exhibit increased osteogenic differentiation and decreased lipogenic differentiation (Wu et al., 2022). Shen J. et al. (Shen et al., 2020)further discovered that miR-483-3p stimulates lipogenic differentiation in ADSCs and inhibits the IGF1 pathway, leading to senescence in ADSCs.

### 2.6 Metabolic alterations

Senescence is closely associated with disturbances in the maintenance of metabolism. As cells become senescent, several neutral amino acids such as valine, isoleucine, and glycine can be utilized as alternative energy sources to maintain energy homeostasis (Yi et al., 2020). Metabolomic analysis has revealed changes in glycerophospholipid metabolism, taurine and hypotaurine metabolism, glycerolipid metabolism, drug metabolism-cytochrome P450, and drug metabolism-other enzymes in senescent BMSCs(Chen et al., 2019; Yu X. et al., 2022). Lipid metabolism was found to be inhibited. Genes related to lipid metabolism, such as Scd, Scd2, Dgat2, Fads2, and Lpin1, were downregulated. Scd2, the most significant differentially expressed gene (DEG), may be involved in altering the biomembrane of senescent cells. In Scd2 overexpressing BMSCs, there was a significant reduction in SA-β-gal activity, and the expression of senescence-associated genes was suppressed. However, there have been limited reports on the roles and mechanisms related to Scd2 (Yu Xiao et al., 2022).

### 2.7 Phenotypic changes

During senescence, there are no significant differences in the expression of certain surface markers such as CD105, CD73, and CD90. However, other surface markers such as CD106, CD146, and STRO-1 are linked to senescence in MSCs and their expressions are downregulated during senescence. For example, MSCs derived from the BM of multiple sclerosis (MS) patients showed senescence-related characteristics such as reduced amplification capability and decreased STRO-1 expression (Redondo J. et al., 2018). The improved effectiveness of CD146+ MSCs in treating myocardial infarction (MI) may be attributed to lower levels of ROS(Zhang et al., 2019). On the other hand, replicative senescence enhances CD26 expression. MSCs with high CD26 levels have a decreased capacity for immunosuppression and proliferation compared to MSCs with low CD26 levels (Psaroudis et al., 2022).

SASPs were initially discovered and described by Coppe et al. in senescent fibroblasts and epithelial cells (Coppe et al., 2008). During senescence, cells produce unique secretions known as SASPs, which play a role in maintaining the senescent phenotype. SASPs consist of various components including inflammatory and immunomodulatory factors (e.g., IL-6, IL-7, and IL-8), chemokines (e.g., MCP-2 and MIP-3a), growth factors (e.g., GRO, HGF, and IGFBPs), cell surface receptors (e.g., ICAMs, uPAR, and TNF receptors), matrix metalloproteinases, and survival factors (Acosta et al., 2008; Acosta et al., 2013; Basisty et al., 2020). In replicative senescent MSCs, there is a significant increase in the levels of SASPs-associated proteins IL-6, IL-8, and MCP-1, with IL-6 showing the highest increase. The development of senescence in MSCs is attributed to enhanced autophagic activity (Bernard et al., 2020), which is closely linked to the upregulation of FOX3a levels and subsequent increase in IL-6 and IL-8 expression (Zheng et al., 2023). However, other SASPs-related proteins such as MIP-1α, MMP-2, MMP-3, IL-1α, and IL-1β were not found to be altered in association with senescence based on relevant studies (Marote et al., 2023).

Researchers have discovered that SASPs promote senescence in MSCs by suppressing B cell-specific Moloney murine leukemia virus Integration site 1 (Bmi-1). The expression of Bmi-1 was found to be downregulated in the senescence environment and reducing Bmi-1 levels decreased the proliferation rate of MSCs. Among the various standard components of SASPs that were tested and compared, IL-1α showed the highest impact on downregulating Bmi-1 (Zheng et al., 2021).

**FIGURE 1:**
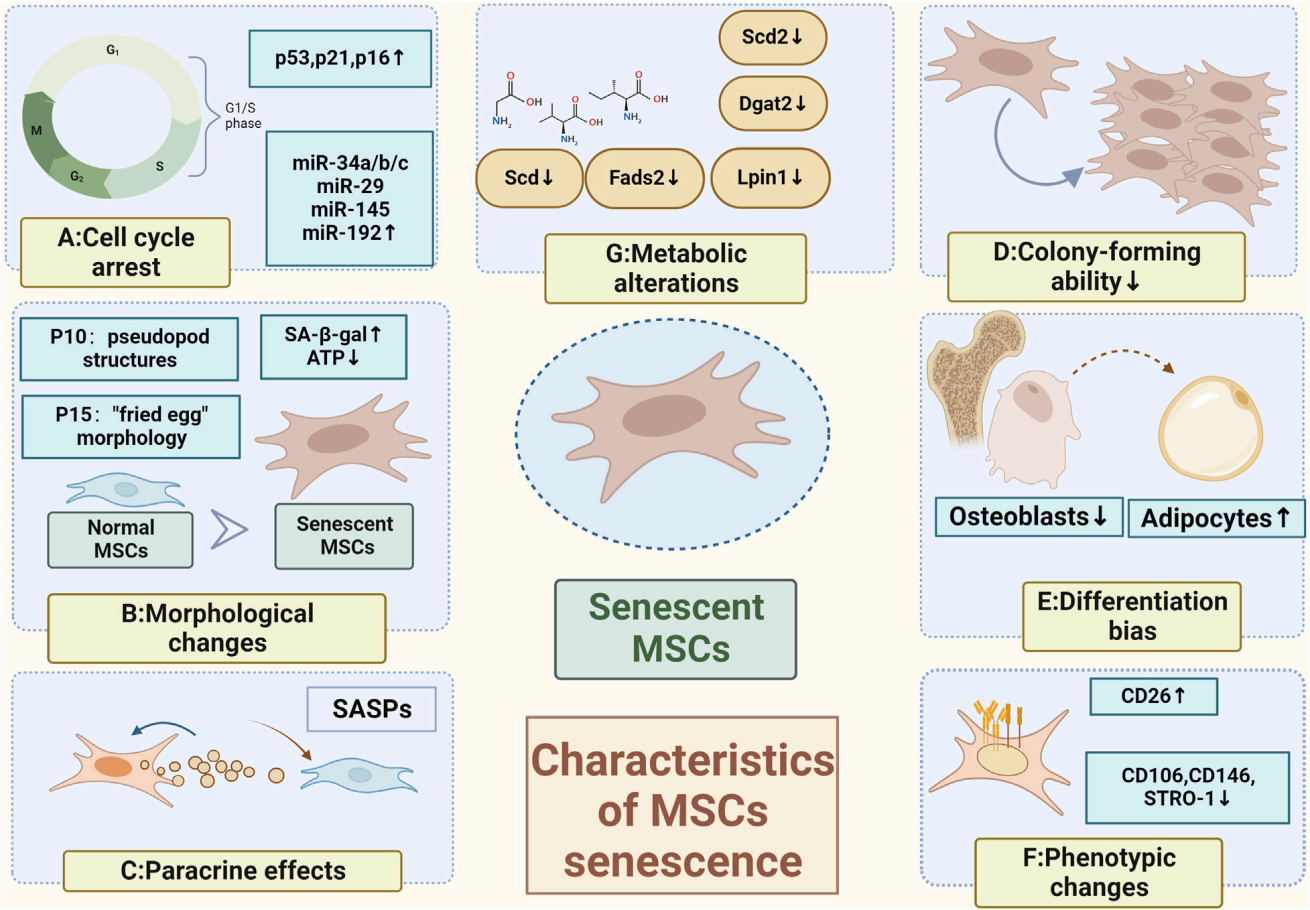
Characteristics of MSCs senescence **(A)** Cell cycle arrest caused by activation of the p53/p21CIP1 signaling pathway or the p16^INK4A/Rb^ oncogenic pathway (Kamal et al., 2020); **(B)** Morphological changes with pseudopod structures appearing in the 10th generation and a "fried egg" morphology in the 15th generation (Truong et al., 2019); **(C)** Paracrine effects; **(D)** Loss of colony-forming abilities (Ridzuan et al., 2016); **(E)** Elevated osteogenic differentiation and a decreased lipogenic differentiation (Yi et al., 2021); **(F)** Phenotypic changes; **(G)** Metabolic alterations (Neutral amino acids such as valine, isoleucine, and glycine can be used as alternative energy sources to maintain energy homeostasis (Yi et al., 2020); Inhibited lipid metabolism (Yu Xiao et al., 2022)) (Created by Biorender. com).

When investigating ways to mitigate senescence in MSCs caused by SASPs, researchers discovered that WNT/β-catenin signaling inhibits the paracrine effects of SASPs. Additionally, Wnt3a was found to have a positive effect on cell proliferation. Furthermore, it was observed that the combination of SASPs inhibitory factors FGF2 and Wnt3a may have a more significant anti-senescent effect (Lehmann et al., 2022). Moreover, in radiation-induced senescent cells, the levels of SASPs factors such as IL-1α, IL-6, MMP-3, resistin, lipocalin, and IGFBP-6 were significantly increased. These factors disrupted the colony-forming ability and multidirectional differentiation of BMSCs through paracrine effects. However, the adverse effects were alleviated when JAK1 inhibitors were used (Xu et al., 2021) (Figure 1).

## 3 Mechanisms of mesenchymal stem cell aging

According to Al-Azab M et al. (Al-Azab et al., 2022), the five main hallmarks of MSC senescence are damage to genetic material, non-coding RNA and exosomes, loss of protein homeostasis, intracellular signaling pathways, and mitochondrial dysfunction. In this section, we will summarize the relevant research progress.

### 3.1 Damage to genetic material

Damage to genetic material can occur through telomere shortening, DNA damage, and epigenetic alterations.

#### 3.1.1 Shortened telomere

Telomeres, which are non-coding regions at the ends of chromosomes, consist of thousands of identical sequence repeats (Blackburn and Gail, 1978; Moyzis et al., 1988). During lagging strand synthesis, DNA polymerase is unable to fully replicate the 3′end of double-stranded DNA (Watson, 1972). Telomere depletion plays a role in regulating the senescence of MSCs through downstream signaling of the oncogene repressor protein p53 and inhibiting mitochondrial metabolic activity via the peroxisome proliferator-activated receptor gamma (PPARγ) coactivator 1α/β (PGC-1α/β) (Sui et al., 2016). MSCs obtained from telomerase knockout animals exhibit impaired replicative capacity and may even lose the ability to differentiate completely, even in early passages (Liu et al., 2004; Pignolo et al., 2008; Yang Y. K. et al., 2018). The average length of telomeres in MSCs is dependent on the age of the tissue donor in early-passage MSCs(Li et al., 2017). Previous research has indicated that the average length of telomeres in early cultures of MSCs ranges from 11–13 to 9–10 kb (Parsch et al., 2004; Bonab et al., 2006). Other studies have shown that during *in vitro* expansion, there is a rapid aging process, resulting in approximately 100 bp of telomere shortening every two passages. Senescence in MSCs is typically observed when the telomere length reaches 10 kb, although a different study reported a length of 6.8 ± 0.6 kb in senescent cells (Oja et al., 2018; Liu J. et al., 2020). The potential of using telomere length measurement as a biomarker in assessing the senescence of MSCs holds significant promise. Telomere shortening stands as one of the best-characterized mechanisms triggering cell senescence, and it can be expedited by the presence of oxidative stress (Jiang et al., 2023). The activation of the catalytic subunit of telomerase (known as hTERT) has been found to effectively impede the progression of senescence, resulting in a notable decrease in aneuploidy levels and the preservation of ploidy-controlling genes' regulation (Estrada et al., 2013). Hence, the close association between telomere length and MSC senescence implies that telomere-based examinations possess the potential to serve as valuable tools in diagnosing and managing cellular senescence (Bernadotte et al., 2016).

#### 3.1.2 DNA damage

DNA damage can result in two outcomes. The first outcome involves the inaccurate repair of DNA damage, which can give rise to mutations or chromosomal aberrations, ultimately culminating in the development of cancer. The second outcome is persistent DNA damage that hinders replication and transcriptional processes, causing cellular dysfunction, cellular senescence, and apoptosis (Ou and Schumacher, 2018; Banimohamad-Shotorbani et al., 2020). Among these outcomes, the accumulation of endogenous ROS plays a crucial role in DNA damage (Canli et al., 2017). As the number of passages increases, the accumulation of ROS and the aggravation of DNA damage can be observed in MSCs. Conversely, the expression of DNA repair-related proteins such as Ku70, Ku80, Rad 51, PAR, and 116 kDa PARP1 decreases with cell passages. The transcription factor PBX1 can mitigate ROS-mediated DNA damage and inhibit the senescence and apoptosis of MSCs(Wang Yuan et al., 2021). Consequently, comprehending the intricate molecular mechanisms driving MSC senescence is imperative to enhance the curative impact of MSCs and create viable approaches to impede or potentially revert the dysfunction of aged MSCs. This, in turn, holds promise for revitalizing individuals' holistic welfare and alleviating age-associated diseases (Weng et al., 2022).

#### 3.1.3 Epigenetic alterations

The cellular senescence of MSCs is orchestrated by various epigenetic modifications, such as the organization of chromatin, posttranslational modifications of histones, DNA methylation, and the involvement of non-coding RNAs(Al Aboud et al., 2023; Sun et al., 2023). In a related study, it was discovered that 46 differentially regulated genes were identified in BMSCs from both young and senescent patients after undergoing *ex vivo* expansion. Out of these genes, 23 were found to be associated with selective shearing (Peffers et al., 2016). Other studies have also reported similar findings, indicating that selective shearing is a characteristic of aging in MSCs. Additionally, differences in methylation levels can influence selective shearing, as senescent MSCs exhibit high levels of hypomethylation or an overall loss of DNA methylation (Bork et al., 2010; Cakouros and Gronthos, 2020).

### 3.2 Non-coding RNA and exosomes

RNA serves as a transmitter of genetic information expression and plays a crucial role as a regulator. Small non-coding RNAs, which have diverse functions in cells, are involved in regulating important life processes such as growth and development, gene expression, genome stability, and cellular senescence. These small non-coding RNAs (SncRNAs) include various types, such as microRNAs (miRNAs), small nuclear RNAs (snRNAs), small nucleolar RNAs (snoRNAs), P-element-induced wimpy testis (PIWI)-interacting RNAs (piRNAs), small interfering RNAs (siRNAs), transfer RNAs (tRNAs), and repeat-associated siRNAs (rasiRNAs). They function as epigenetic regulatory molecules in the regulation of cellular senescence in MSCs(Musavi et al., 2019; Wang S. et al., 2021).

BMSCs were amplified *ex vivo* and exhibited replicative senescence, as evidenced by increased SIRT1 mRNA expression and significantly increased SA-β-gal activity. Additionally, there were observed differences in gene expression of SncRNAs, including 203 miRNAs, 46 piRNAs, 63 snoRNAs, 12 snRNAs, and 7 rasiRNAs in p10 generation BMSCs compared to p1 generation BMSCs(Xiao et al., 2022).

miRNA is a crucial component of exosomes, which are a type of EVs that plays a dual role in anti-aging and senescence. It induces senescence when it is a part of SASPs, but resists senescence when secreted by young and healthy MSCs(Ahmadi and Rezaie, 2021). The imbalance between mitochondrial fusion and fission is closely related to cellular senescence. Liangge He et al. proposed that miR-311 derived from EVs could be an important indicator of senescence promotion for diagnosing inflammation and acute senescence in MSCs(He et al., 2023). Dynamin-related protein 1 (Drp1) mediates mitochondrial fission, resulting in the formation of small, round mitochondria. On the other hand, miR-155-5p derived from senescent MSCs induces mitochondrial fusion and drives normal MSCs into senescence by inhibiting the Cab39/AMPK signaling pathway. The senescence of MSCs can be alleviated by using miR-155-5p inhibitors, although this effect can be partially reversed by the Drp1 inhibitor Mdivi 1 (Hong et al., 2020). Furthermore, Nampt plays a key role in the regulation of natural and replicative senescence in MSCs through the inhibition of NAD-SIRT1 signaling (Ma et al., 2017). The expression of miR-34a increases in senescent MSCs and directly suppresses Nampt, thereby mediating the induction of MSC senescence (Pi et al., 2021).

### 3.3 Dysregulation of protein homeostasis

Protein homeostasis, the balance between protein synthesis and degradation, is regulated by a protein quality control system comprising molecular chaperones, ubiquitin proteasomes, and cellular autophagy. Additionally, the regulation of these signaling pathways involves mTOR pathways, Hippo signaling, and Rank signaling (Kapetanou Marianna et al., 2017). During aging, impairment of cellular function is closely associated with the imbalance of protein homeostasis, with the loss of proteasome function playing a central role in this process. Late-passing hMSCs exhibited senescence-associated proteasomal alterations, including reduced mRNA and protein expression of several characteristic proteasomal subunits (such as β1, β2, β5, α4, α7, and Rpt6), as well as decreased 26 S proteasome activity and increased 20 S proteasome activity (Chondrogianni et al., 2005; Kapetanou M. et al., 2017).

### 3.4 Mitochondrial dysfunction

Mitochondria in senescent cells undergo various changes, including altered metabolic function (Seok et al., 2020), increased production of ROS, higher mitochondrial mass, decreased membrane potential (Korolchuk et al., 2017), and subsequent acceleration of telomere shortening and DNA damage (Chapman et al., 2019).

Mitochondria function as the cell’s energy factory, producing ATP and metabolic energy sources through oxidative phosphorylation (OXPHOS). However, the overproduction of ROS by complexes I and III of the respiratory chain can lead to cellular senescence in cases of mitochondrial failure (Ray et al., 2012). Mitochondrial autophagy plays a vital role in eliminating damaged or dysfunctional mitochondria. One example of its impact is the varying effect of H_2_O_2_ on mitochondrial autophagy, where prolonged exposure inhibits the process and leads to apoptosis in BMSCs. This inhibition is associated with the suppression of Jun N-terminal kinase (JNK), a member of the mitogen-activated protein kinase (MAPK) family (Fan et al., 2019). Another instance is the protective effect of curcumin (Cur) on cellular autophagy, which is essential for maintaining cellular homeostasis and controlling the senescence of MSCs (Deng et al., 2021).

### 3.5 Intracellular signaling pathways

#### 3.5.1 IGF-1 pathway

Insulin-like growth factor 1 (IGF-1) plays a crucial role in processes such as growth and lipid metabolism. Previous research suggests that when IGF-1 binds to its receptor, it can alleviate senescence in MSCs. This effect is associated with the activation of the PI3K/Akt pathway (Tian et al., 2020). The phosphorylation of AKT and the high expression of SFRP2 further activate the Wnt/β-catenin pathway, which helps maintain the cellular proliferative capacity and metabolic functions of MSCs(Lin et al., 2020). Additionally, other studies have demonstrated that IGF-1 inhibits its downstream effector proteins (p70S6K and S6) through the Akt/mTOR pathway. This, in turn, increases cellular autophagy to prevent MSCs from undergoing apoptosis (Yang Ming et al., 2018).

#### 3.5.2 mTOR pathway

Persistent activation of the growth-promoting mammalian target of rapamycin (mTOR) pathway has been shown to play a central role in cellular senescence and individual aging (Johnson et al., 2013). Inhibition of the mTOR pathway facilitates the delay of replicative senescence and the maintenance of cell stemness in MSCs(Antonioli et al., 2019). The protective mechanism mainly involves preventing the accumulation of intracellular ROS and DNA damage, as well as reducing the expression of senescence-associated inflammatory cytokines and genes (e.g., p16^INK4A^). Increasing the level of cellular autophagy through selective inhibition of mTORC1 is an effective way to slow down MSC senescence. The combination of AMPK activator (5-aminoimidazole-4-carboxamide ribonucleotide, AICAR) and SIRT1 activator (nicotinamide, NAM) inhibits mTORC1 activity and delays MSC senescence. AICAR not only enhances the level of autophagy and maintains the morphological and proliferative capacity of MSCs but also preserves mitochondrial homeostasis through the activation of SIRT1 by AMPK, thereby reducing ROS and increasing the level of the anti-apoptotic gene Bcl-2 (Khorraminejad-Shirazi et al., 2020).

There are three Hedgehog proteins in mammals: sonic hedgehog (Shh), Indian hedgehog (Ihh), and desert hedgehog (Dhh). These proteins are highly conserved across evolution and species and have significant roles in the development of skeletal tissue (Briscoe Therond, 2013). In a separate study investigating the impact on cell cycle pathways in MSCs, Al-Azab et al. (Al-Azab et al., 2020) discovered that the Ihh, hinders the progression of the ROS/PI3K/Akt/NF-B/mTOR/4EBP1-p70S6K pathway. Furthermore, the study revealed that when Ihh expression is suppressed, the cell cycle of MSCs is arrested and then enters a state of senescence.

#### 3.5.3 AMPK pathway

AMP-activated protein kinase (AMPK) is a crucial regulator of cellular and organismal energy metabolism. Its activation depends on the energy status and the activity of upstream stimulatory and inhibitory signaling pathways. However, with aging, AMPK’s reactivity declines, and its regulatory capacity diminishes (Salminen et al., 2016). FGF21, on the other hand, can regulate mitochondrial survival by modulating AMPK activation. This regulation involves increasing protein levels of p-AMPK and p-Drp1, reducing intracellular ROS levels, and delaying senescence in BMSCs (Li et al., 2019). Resveratrol also promotes osteogenic differentiation and delays aging in BMSCs through the AMPK/ROS signaling pathway (Zhou et al., 2019).

#### 3.5.4 NF-κB pathway

The NF-kB family consists of five transcription factors (p50, p52, p65, c-REL, and ReIB) that are closely associated with immune and inflammatory responses (Kim et al., 2019). Numerous data indicate a strong correlation between p65, SASPs, and their paracrine effects. Similar levels of p65, IL-6, and IL-8 were observed in both DNA damage-induced MSCs senescence (DDIS) and treatment-induced senescence (TIS), as well as in a pro-inflammatory activation (PA) model in MSCs using TNF-α. This suggests that p65 pathway activation occurs during cellular senescence and pro-inflammatory activation in MSCs. Additionally, p65 can enhance the release of small extracellular vesicles (sEV) by MSCs, which in turn promotes peripheral cellular aging through a paracrine pathway (Banimohamad-Shotorbani et al., 2020; Mato-Basalo et al., 2021).

#### 3.5.5 Sirtuins pathway

Sirtuins, a family of NAD-dependent deacetylases, consist of seven members known as SIRT1–7. These sirtuins have shown high conservation throughout evolution (Almeida and Porter, 2019). They play crucial roles in various cellular activities such as cellular autophagy, metabolism, DNA repair, apoptosis, and cellular senescence. Among them, SIRT1 is a significant target for extending lifespan and delaying senescence as it integrates multiple signaling and transcriptional pathways. Some of the known pathways involved in this process include the p65-NF-κB pathway, the p53-DNA damage pathway, the mTOR-cellular autophagy pathway, the AMPK pathway, the FOXO-DNA damage and oxidative stress pathway, and the PGC1α-mitochondrial autophagy pathway (Chen Cui et al., 2020). SIRT3, a mitochondrial sirtuin, is involved in various metabolic regulations. Studies have shown that SIRT3 upregulates the expression and activity of superoxide dismutase 2 (SOD2), which helps inhibit premature senescence in MSCs induced by natural and oxidative stress (Ma et al., 2020). On the other hand, senescent MSCs exhibit downregulation of the NAD/SIRT3 signaling pathway. To counteract this, supplementation with nicotinamide mononucleotide (NMN), a precursor of NAD, can upregulate the NAD/SIRT3 signaling pathway in replicative senescent MSCs. This supplementation improves mitochondrial function and rescues MSCs from senescence (Wang Huan et al., 2022).

## 4 Effects of different cellular microenvironments on MSCs' senescence and functions

### 4.1 Aging microenvironment

The senescent microenvironment is defined by several key features: impaired fibroblast function, an accumulation of senescent fibroblasts, disruption of the extracellular matrix’s integrity, and the initiation of age-related chronic inflammation (Fane and Weeraratna, 2020; Wang X. et al., 2022; Ye et al., 2023).

The SASPs play a crucial role in the formation of the senescent microenvironment. While senescent cells and SASPs can have temporary beneficial effects, such as improving the regeneration and stemness of keratin-forming cells through brief exposure to SASPs, they can become problematic as the body ages. Senescent cells tend to accumulate in the body tissues and cause harm to the organism (Ritschka et al., 2017). Research has demonstrated that the existence of SASPs is linked to the emergence of degenerative diseases and cancer. Furthermore, SASPs contribute to chronic inflammation and hinder tissue repair functions. Another detrimental effect of SASPs is their paracrine influence, wherein neighboring cells are transformed into senescent cells through paracrine action (Xu et al., 2018; Al Suraih et al., 2020; Kowald et al., 2020). Eliminating senescent cells can decrease the production of SASPs, leading to a better prognosis for geriatric syndromes and aging-related diseases, as well as an enhanced organism repair capacity (Pignolo et al., 2020).

Extracellular vesicles (EVs) are significant components of the senescence microenvironment (Yin Y. et al., 2021). Mesenchymal stem cell-derived extracellular vesicles (MSC-EVs) are considered a promising therapeutic tool for immunomodulation and regeneration. These vesicles, which are nano-sized and enclosed by a membrane, contain important biomolecules such as mRNAs, microRNAs, bioactive lipids, and signaling proteins (Massa et al., 2020). The advantages of MSC-EVs over MSCs include a higher safety profile, lower immunogenicity, and the ability to traverse biological barriers (Varderidou-Minasian et al., 2020). Furthermore, the use of MSC-EVs helps avoid complications associated with stem cell-induced ectopic tumor formation, entrapment in lung microvasculature, and immune rejection. However, there are still challenges and barriers to the clinical translation of MSC-EVs, such as quality control and efficiency (Huang et al., 2019b; Weng et al., 2021). Additionally, as part of SASPs, MSC-EVs play a crucial role in promoting cellular senescence due to their paracrine effect and messaging function (Sun Zixuan et al., 2022). It is worth investigating further whether senescent MSC-EVs may have different effects depending on the tissue, age, or context (such as inflammation or disease). EVs derived from aged myoblasts have been discovered to induce senescence in primary BMSCs in an *ex vivo* setting (Jiang et al., 2023). Furthermore, studies have demonstrated that both circulating and tissue-derived aged EVs can induce senescence in MSCs cultured *ex vivo* (Weilner et al., 2016; Davis et al., 2017).

**FIGURE 2:**
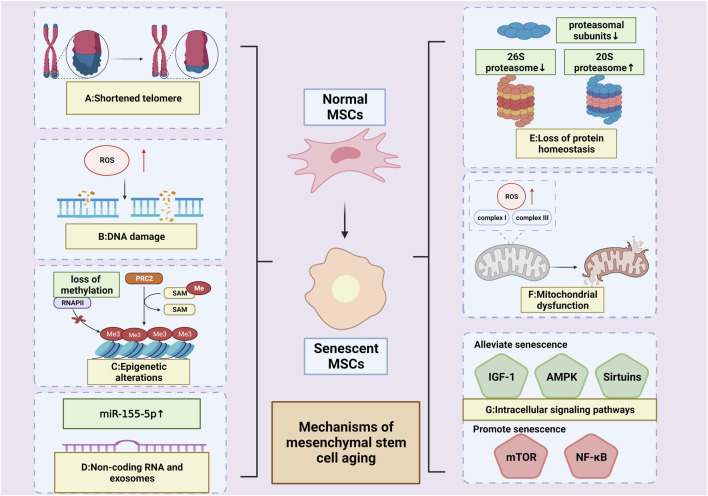
Mechanisms of MSCs’ aging. **(A)** Shortened telomere; **(B)** DNA damage; **(C):** Epigenetic alterations (High levels of hypomethylation or an overall loss of DNA methylation (Cakouros et al., 2020; Bork et al., 2010)); **(D)**: Non-coding RNA and exosomes (Upregulated miR-155-5p (Hong et al., 2020)) **(E)** Loss of protein homeostasis (reduced proteasomal subunits; decreased 26 S proteasome activity; increased 20 S proteasome activity (Chondrogianni et al., 2005; Kapetanou M. et al., 2017)); **(F)**:Mitochondrial dysfunction (overproduction of ROS by complexes I and III of the respiratory chain (Ray et al., 2012));**(G)**: Intracellular signaling pathways (pathways alleviating senescence: IGF-1,AMPK&SIRT;pathways promoting senescence: mTOR&NF-κB). (Created by Biorender. com).

MSC therapies have shown promising prospects for applications in cellular and animal experiments. However, the current clinical outcomes are not satisfactory. Some scholars speculate that the age of the patient may contribute to this situation (Figure 2). This is because, while most cell and animal experiments involve young individuals as donors and patients, in clinical practice, the elderly are the primary recipients of MSCs treatment. (Chen Huan et al., 2022).

With aging, elderly individuals often experience a chronic, low level of subclinical proinflammatory state (Chung et al., 2011; Lin et al., 2017). This is characterized by an increase in the expression of proinflammatory cytokines and chemokines in their serum. Specifically, the proinflammatory cytokine IL-6 shows a noticeable increase, while the anti-inflammatory cytokine IL-10 decreases significantly. The elevated levels of IL-6 trigger the activation of JAK/STAT and MAPK pathways, which further contribute to the aging of MSCs (Peng et al., 2022). Additionally, studies have shown that MSCs cultured in the extracellular matrices (ECM) of elderly individuals also exhibit reduced proliferation potential of BMSCs. This impairment is closely associated with a decrease in Cyr61/CCN1. However, the exogenous addition of Cyr61 has been found to help restore the ECM’s response to IGF-1 signal (Marinkovic et al., 2022).

In addition to the aging of the treated patients themselves, the extraction of MSCs from aged individuals with premature aging properties significantly reduces the efficacy of the treatment. (Yamaguchi et al., 2018).

MSCs derived from aged individuals typically display the following characteristics: an elevated number of senescent cells, heightened expression of senescent genes and proteins (such as p21 and γH2AX), increased SA-β-gal activity, and reduced capacity for osteogenic, lipogenic, and angiogenic differentiation (Liu et al., 2017; Chen Xihang et al., 2021). The senescent microenvironment promotes premature aging and impairs the differentiation ability of MSCs through several mechanisms. These include upregulation of CD137 expression, inhibition of Bcl-3 expression (Wang F. X. et al., 2022), and disruption of Wnt/β-catenin signaling pathway transmission (Han et al., 2022).

BMSCs derived from aged individuals displayed impaired cell migration function and downregulation of genes involved in cell motility, such as DPP4, Egf, Actn3, Rho, Cav1, *etc.* Additionally, the reduced number of CD90^+^ BMSCs was associated with impaired wound healing ability (Amini-Nik et al., 2022).

Metabolic dysfunction of BMSCs was found to be positively correlated with senescence. In BMSCs from senescent mice, the expression levels of critical enzymes for mitochondrial genesis and glycolysis were reduced, resulting in impaired oxidative phosphorylation and glycolytic function. Consequently, this affected the stemness and differentiation potential of BMSCs(Li et al., 2022).

The adverse effects of MSCs from aged individuals during treatment are linked to their impaired macrophage recruitment. This impairment is attributed to altered expression of miRNAs that regulate this process. Specifically, miR-223-5p expression is downregulated, while miR-127-3p and miR-125b-5p expression are increased (Huang et al., 2019a).

**FIGURE 3:**
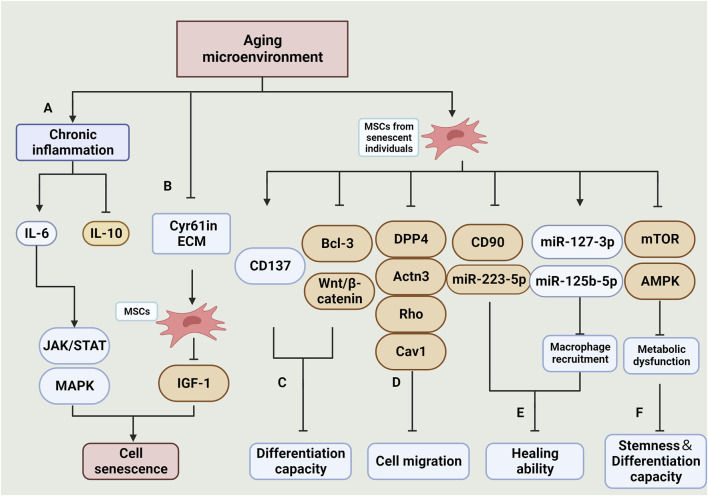
Effects and mechanisms of the aging microenvironment on MSCs. **(A)**: Chronic inflammation in the aging microenvironment results in an increase in pro-inflammatory factors, primarily IL-6, and a decrease in anti-inflammatory factors, primarily IL-10. The elevated IL-6 activates the JAK/STAT and MAPK signaling pathways, ultimately leading to the senescence of MSCs (Peng et al., 2022). **(B)**: Reduction of Cyr61 in ECM decreases the sensitivity of MSCs to IGF-1 signalling, thereby increasing their vulnerability to senescence (Marinkovic et al., 2022). **(C):**MSCs derived from senescent individuals exhibit impaired differentiation capacity in culture. This is primarily attributed to increased CD137 expression, decreased Bcl-3 expression, and inhibition of the Wnt/β-catenin pathway (Wang F. X. et al., 2022) (Han et al., 2022). **(D)**: The reduction in expression of DPP4, Actn3, Rho, Cav1, and other related genes is associated with impaired cell migration (Amini-Nik et al., 2022). **(E)**: The impaired therapeutic efficacy of MSCs is linked to the increased expression of CD90 and miR-223-5p, as well as the impaired recruitment of macrophages due to the decreased expression of miR-127-3p and miR-125b-5p (Huang et al., 2019a). **(F)**: Inhibition of the mTOR and AMPK signaling pathways results in impaired cellular metabolic function, ultimately leading to senescence in MSCs(Li et al., 2022). (Created by Biorender. com).

The presence of a senescent microenvironment leads to chronic inflammation, which in turn significantly reduces the number and function of MSCs (Brunet et al., 2023). In the field of regenerative medicine, senotherapy research is currently focused on interventions that target the MSCs microenvironment and senescent stem cells during *in vitro* expansion. This research includes studying senolytics and senomorphic mechanisms and targets, exploring optimal therapeutic doses and routes of administration, as well as selecting and applying specific drugs (Wong et al., 2023) (Figure 3).

### 4.2 Hypoxic/ischemic microenvironment

*In vivo*, MSCs are commonly exposed to physiological conditions, encompassing oxygen concentrations that span from 2% to 8%.The oxygen levels typically observed in culture, around 18.4% O2, can be deemed as severely hyperoxia for MSCs when compared to their original niches (Phelps et al., 2023). Previous studies have examined the impact of hypoxic microenvironments on the senescence of MSCs, categorizing them into two groups: 'physiological hypoxia', which supports MSC survival (Buravkova et al., 2014), and 'pathological hypoxia', which accelerates senescence and apoptosis of MSCs. It is worth noting that the pathological hypoxic microenvironment is often accompanied by a lack of blood perfusion, further compromising cell survival.

Hypoxic preconditioning typically involves incubation at (1%–5%)O_2_ for 48 h or 72 h, as compared to physiological conditions (21% O_2_). Existing studies collectively indicate that hypoxic preconditioning effectively reverses the senescent state of MSCs primarily by modulating the cellular autophagy axis and enhancing intracellular ROS levels. Hypoxia preconditioning increased the expression level of HIF-1α in BMSCs, thereby enhancing cell viability and reducing the expression of the apoptotic protein caspase3, which leads to an increase in the transplantation viability of BMSCs under oxidative stress conditions (Luo et al., 2019). The improved survival rate of BMSCs after hypoxic pretreatment was attributed to the upregulation of survival-related genes LPL, PKM, and MAP3K13(Peck et al., 2021). According to Kim et al. (Kim et al., 2019) their study revealed that AIMP3 plays a crucial role in delaying the senescence of MSCs under hypoxic conditions. They also found that overexpression of AIMP3 inhibits cellular autophagy, which leads to senescence and dysfunction of MSCs. Additionally, the hypoxic microenvironment enhances the angiogenic capacity of BMSCs by increasing the expression of VEGF, a factor downstream of HIF-1α. Furthermore, BMSCs exhibit increased expression of osteogenic-related genes (RUNX2 and OCN) and enhanced potential for osteogenic differentiation (Zhang et al., 2018). Liu et al. (Liu Wei et al., 2020) discovered that culturing BMSCs under hypoxic conditions resulted in the increased release and secretion of exosomes and exosome-related proteins (TSG101, CD9, CD63, and CD81). Furthermore, they found that MSC-EVs under hypoxic conditions could effectively deliver miR-216a-5p to microglia. This delivery triggered a cascade reaction involving TLR4/NF-κB/PI3K/AKT, leading to the conversion of microglia from M1 to M2 type and subsequently reducing the associated inflammatory damage.

In contrast to the appropriate physiological hypoxic microenvironment described above, a pathological hypoxic (and ischaemic) microenvironment would undoubtedly lead to premature senescence and apoptosis of MSCs.

Systemic chronic hypoxia is a pathological state characterized by insufficient systemic oxygen supply. It is closely linked to conditions such as cyanotic congenital heart disease (CCHD), chronic obstructive pulmonary disease, chronic mountainous disease, and the development of pulmonary fibrosis. (Xing et al., 2018). Rehman SU et al. (Rehman et al., 2017) utilized patients with CCHD as a human disease model of chronic systemic hypoxia to investigate the conditions and the role of BM in association with gut microbes, and its effect on BMSCs aging. The study revealed an association between this phenomenon and an imbalance in intestinal ecology and the metabolism of d-galactose by the intestinal microbiota. Cellular senescence could be induced by d-galactose through the production of large amounts of ROS. Additionally, a negative correlation was observed between the concentration of d-galactose and the number of lactobacilli in the intestine. Upon administering appropriate amounts of *Lactobacillus*, the accumulation of d-galactose in rats was reduced, and the deficiency of BMSCs was significantly restored.

While stem cell transplantation has been shown to aid in cardiomyocyte repair following acute myocardial infarction (AMI) (Miao et al., 2017), it is important to note that the survival and differentiation of MSCs can be influenced by the local microenvironment. In particular, the ischemic and hypoxic microenvironment resulting from AMI can lead to a low survival rate of MSCs post-transplantation (Khodayari et al., 2019). Qi Y et al. (Qi et al., 2021) investigated the impact of hypoxic and serum deprivation (H/SD) conditions on the microenvironment of acute myocardial infarction (AMI). They observed that under H/SD conditions, the viability and migration of BMSCs were reduced compared to the normal control group. They also found an increase in apoptotic cells and the expression of apoptosis-related proteins Bax and cleaved-caspase3. This effect was attributed to the increased presence of M1-type macrophages and the upregulation of M1-type macrophage factors TNF-α and IL-1 in the H/SD condition. The secretion of TNF-α and IL-1 further increased, and M1-type macrophage exosomes inhibited the expression of Bcl-2 protein and induced apoptosis in BMSCs through miR-222. Moreover, BMSCs cultured in hypoxic ischemic (HI) environments exhibited reduced cell migration capacity compared to the normal controls. These cells also showed impaired cell proliferation, possibly due to the inhibition of the PI3K/AKT pathway (Chen Xuxiang et al., 2022).

Ischemia/reperfusion (I/R) injury is a significant contributor to tissue dysfunction, which often leads to organ transplant failure. When blood flow is interrupted, the kidney experiences hypoxia, increased oxidative stress, and microvascular dysfunction (Soares et al., 2019). Additionally, I/R kidney tissue shows elevated levels of ROS and reduced expression of VEGF in MSCs(Bai et al., 2018; Najafi et al., 2022).

**FIGURE 4:**
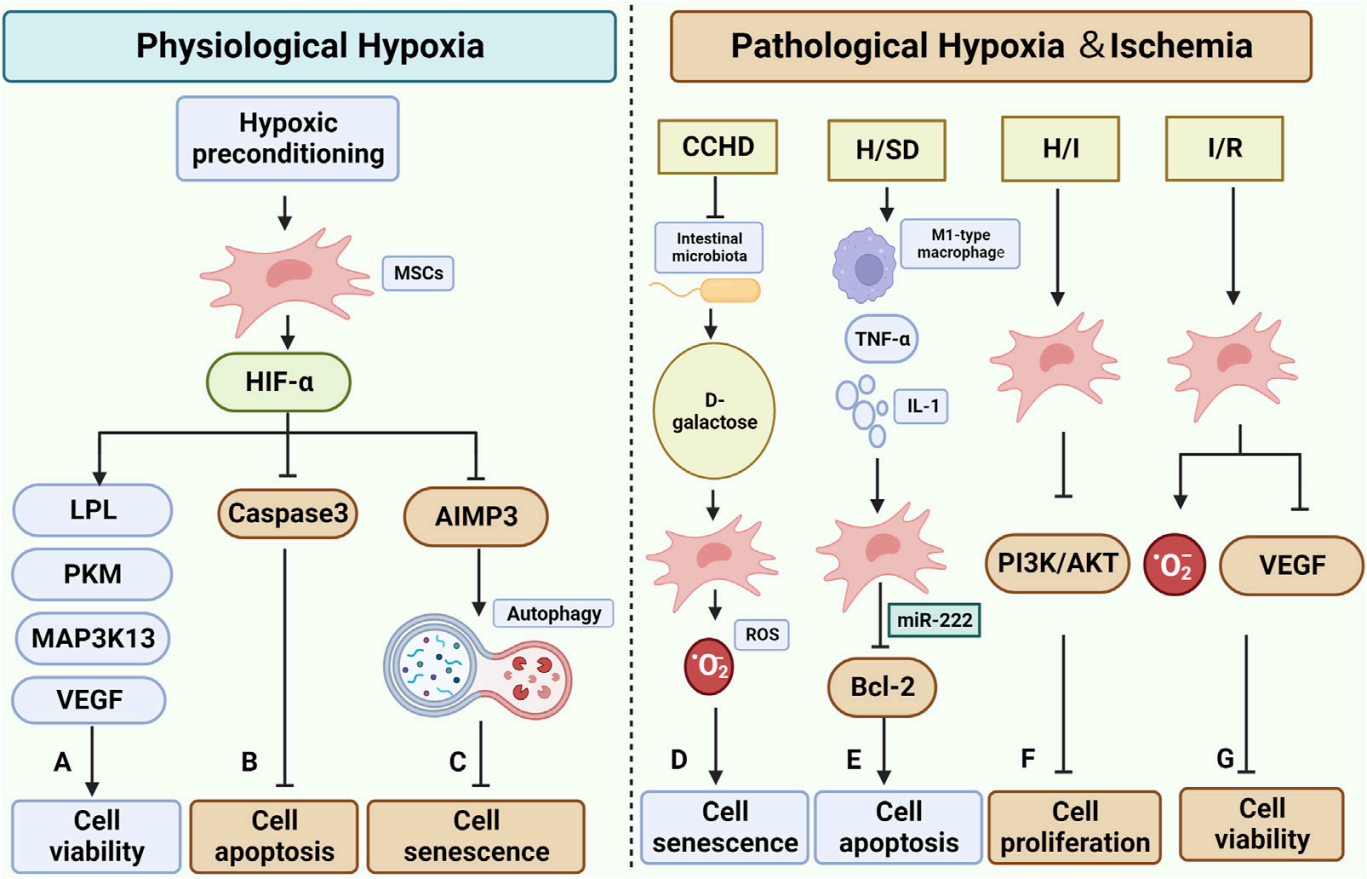
Effect and mechanisms of physiological and pathological hypoxic microenvironment on MSCs. **(A–C)** are the effects of normal physiological hypoxia on MSCs, mostly positive. Hypoxic preconditioning has been shown to impact the function of HIF-α in MSCs. **(A)** It increases cell survival by up-regulating the expression of LPL, PKM, MAP3K13, and VEGF(Peck et al., 2021) (Zhang et al., 2018). **(B)** It inhibits apoptosis by suppressing the expression of Caspase3(Luo et al., 2019). **(C)**: It inhibits the expression of AIMP3, which helps maintain cellular autophagy and consequently slows down cellular senescence (Zhang et al., 2018). D-G in the figure illustrate the impact of pathological hypoxic/ischemic microenvironment on MSCs. **(D)** Patients with CCHD undergo a reduction in Intestinal microbiota, leading to an increase in D-galactose. This increase in D-galactose can elevate the level of ROS in MSCs, contributing to cellular senescence (Rehman et al., 2017). **(E)** The H/SD microenvironment induces the increase of M1-type macrophage and the related regulatory factor TNF-α and IL-1, while decreasing the secretion of miR-222 from MSCs. This downregulates Bcl-2 and promotes apoptosis (Qi et al., 2021). **(F)** The H/I microenvironment hinders the PI3K/AKT pathway in MSCs, thereby suppressing cell proliferation (Chen Xuxiang et al., 2022). **(G)** MSCs in I/R encounter elevated ROS levels and downregulation of VEGF, resulting in decreased survival (Bai et al., 2018; Najafi et al., 2022) (Created by Biorender.com).

In the context of the pathological hypoxic and/or ischaemic microenvironment, there are two primary mechanisms through which it negatively affects MSCs. Firstly, it regulates the cellular metabolic capacity. Secondly, it exposes the cells to pro-inflammatory factors such as TNF-α, IL-1β, and IL-6 for an extended period. This prolonged exposure to pro-inflammatory factors leads to apoptosis, necrosis, and autophagic cascades (Venkatachalam et al., 2009; Oka et al., 2012). Future research could focus on exploring the targets of hypoxic preconditioning to enhance the survival of MSCs. Additionally, investigating the effects of combining hypoxic preconditioning with other drugs could be a potential direction for further study (Figure 4).

### 4.3 Microenvironment of immune diseases

Due to their immunomodulatory and tissue repair effects, MSCs are considered valuable tools for treating immune diseases (Yang et al., 2023). However, various studies have revealed that MSCs in the microenvironment of immune diseases undergo senescence-related alterations. These alterations include reduced cell proliferation capacity, increased expression of p53 and p16, elevated levels of ROS and DNA-damage-response (DDR), and impaired differentiation capacity. These changes are likely associated with the persistent pro-inflammatory microenvironment created by immune diseases. In the subsequent discussion, we will explore the effects and underlying mechanisms of different immunological diseases on MSC senescence and function.

#### 4.3.1 Systemic lupus erythematosus (SLE)

SLE is a chronic autoimmune disease characterized by an imbalance in T cell ratios (Wieliczko and Matuszkiewicz, 2017). The progression of SLE is exacerbated by an imbalance between Follicular helper T cells (Tfh) and regulatory T cells (Treg) (Lee, 2018). Additionally, the pathogenesis of SLE involves a deficiency of the cytokine IL-2, which is a crucial growth and survival factor for Treg cells that play a vital role in controlling autoimmunity in SLE (Humrich and Tiemekasten, 2016). Chen X et al. (Chen Xinpeng et al., 2021)discovered increased levels of LncRNA H19 in the serum and BMSCs of SLE patients. This increased level inhibited the proliferation and migration of BMSCs and promoted their apoptosis. Furthermore, lncRNA H19 impaired the immune properties of BMSCs by reducing the expression level of IL-2, thus inhibiting the differentiation of Treg cells, and disrupting the balance between Treg cells and Tfh cells. Immunomodulatory dysfunction in SLE-derived BMSCs is linked to the decrease in let-7f expressions. Research has shown that reduced expression of Let-7f leads to a decrease in the number of Th17 cells, an increase in the number of Treg cells, and an elevated rate of apoptosis in BMSCs. This effect is achieved through the activation of the STAT3 pathway by IL-6. (Geng et al., 2020).

In addition to altered immunomodulatory functions, hBMSCs derived from patients with SLE exhibited increased expression of genes related to SASP and pro-inflammatory cytokines. This upregulation was mediated by the MAVS-IFNβ axis and resulted in cellular senescence. The activation of the JAK-STAT signaling pathway in SLE-derived BMSCs was also found to be closely associated with premature cellular senescence (Gao and Bird, 2017; Ji et al., 2017). Furthermore, studies have demonstrated that the inflammatory factor HMGB1 in the SLE bone marrow microenvironment can induce senescence in MSCs through the TLR4/NF-κB signaling pathway. However, the HMGB1 inhibitor ethyl pyruvate (EP) can inhibit HMGB1 and improve lupus nephritis, leading to a reversal of senescence in MSCs (Ji et al., 2019).

#### 4.3.2 Ankylosing spondylitis (AS)

AS is a common rheumatic disease that affects approximately 0.1%–0.5% of the global population (Ward et al., 2019). MSCs derived from AS patients exhibit an abnormally increased capacity for osteogenesis, leading to pathological osteogenesis and subsequent bone formation (Liu et al., 2019; Ye et al., 2019). The downregulation of Dickkopf-1 (DKK-1) expression, a crucial regulator of bone remodeling in spondyloarthropathies, is observed in MSCs from AS patients. This downregulation is believed to be caused by IL-17-mediated DKK-1 downregulation, resulting in the activation of the Wnt pathway and upregulation of osteogenesis-related genes (RUNX2, OSX, and ALP) (Daoussis et al., 2022).

The serum microenvironment of patients with AS exhibits abnormal levels of inflammatory factors (such as TNF-α and IL-17) and oxidative stress (Zeng et al., 2011). Among these factors, Advanced Oxidative Protein Products (AOPPs), which are markers of oxidative stress, are associated with disease activity and show a positive correlation. AOPPs can induce the production of ROS and lead to cell cycle arrest. Therefore, it is hypothesized that targeting AOPPs could be a key approach for treating AS-induced aging of MSCs(Karakoc et al., 2007; Sun et al., 2018; Ye et al., 2020).

#### 4.3.3 Inflammatory bowel disease (IBD)

IBD is characterized by Crohn disease (CD) and ulcerative colitis (UC) (Chang, 2020). MSCs derived from CD are more severely impaired in their ability to differentiate and cannot form cell colonies compared to MSCs derived from UC(Grim et al., 2021).

The senescence of MSCs is associated with the onset of inflammation in the lesion area. The presence of pro-inflammatory cytokines (such as INF-α, TNF-α, and IL-6) and paracrine effects lead to premature senescence of MSCs and hinder their differentiation into enterocytes. (Onyiah and Colgan, 2016; Wang et al., 2020). Currently, the low survival rate of stem cells is a pressing issue that needs to be addressed. Several approaches have shown promise in improving the success rate of treatment, including combining stem cell therapy with surgical treatment, orally delivering stem-cell-loaded hydrogel microcapsules (SC-HM), and utilizing EVs(Panés et al., 2018; Alpdundar Bulut et al., 2020; Kim et al., 2022).

#### 4.3.4 Multiple sclerosis (MS)

MSCs derived from patients with multiple sclerosis (MS) exhibit impaired expansion in vitro culture, as well as accelerated rates of senescence and telomere loss. These changes are linked to a reduction in antioxidant capacity, as MS patient-derived MSCs demonstrate decreased secretion of the antioxidants superoxide dismutase 1 (SOD1) and glutathione S-transferase *p* (GSTP). Additionally, there is a decrease in the expression levels of Nrf2 and PGC1α, which regulate the secretion of SOD1 and GSTP. (Redondo et al., 2018b; Redondo et al., 2018c).

#### 4.3.5 Neuromyelitis optica (NMO)

MSCs derived from NMO exhibit impaired proliferative capacity and increased susceptibility to senescence. This is accompanied by a significant upregulation of the pro-apoptosis-related gene Fas and a significant downregulation of the pro-survival gene Bcl-xl. Platelet-derived growth factor (PDGF) plays a crucial role in stimulating the proliferation of MSCs and has shown potential as a cytokine for treating demyelinating diseases. Specifically, PDGF-BB, the primary growth factor found in bone matrix (Chen et al., 2015), has been found to effectively enhance MSC proliferation and counteract premature senescence (Yang et al., 2019). Further research should investigate the specific target and conditions of PDGF-BB’s action for potential therapeutic applications in MSC treatment.

#### 4.3.6 Pulmonary fibrosis

BMSCs obtained from patients with idiopathic pulmonary fibrosis exhibit senescence characteristics, including reduced proliferation, trilineage differentiation, and migration capacity. These characteristics have been linked to the activation of the NADH-AMPK-p53 regulatory pathway, which results in mitochondrial dysfunction in BMSCs. Additionally, the paracrine effects of these cells induce premature senescence in lung fibroblasts. (Cardenes et al., 2018). The aforementioned study indicates that enhancing the secretory function of MSCs is a crucial aspect in the treatment of Pulmonary fibrosis conditions.

### 4.4 Hyperglycemic microenvironment

Hyperglycemia, oxidative stress, and altered immune responses are prominent features of the diabetic microenvironment. Previous studies have shown that these factors contribute to the senescence of MSCs. (Yin Min et al., 2021). hWJSCs obtained from pregnant women with gestational diabetes mellitus (GDM) exhibit impaired osteogenic and chondrogenic differentiation capacity. Additionally, these hWJSCs show downregulated levels of stemness markers, telomerase, antioxidant enzymes, and mitochondrial functional gene expression (ND2, TFAM, PGC1α, and NDUFB9). Moreover, there is an increase in cell cycle arrest-related factors (p16, p21, p27) and senescence-related gene p53 in these MSCs. It is worth noting that their lipogenic capacity remains unaffected (Kong et al., 2019).

The mechanism of hyperglycemia induced MSCs aging primarily involves a shift in the metabolic pattern of MSCs from glycolysis to oxidative phosphorylation. This shift leads to the excessive accumulation of ROS and DNA damage, activating the p53-p21-pRB axis (Wu, 2021). Consequently, telomerase is inactivated, and mitochondrial function is impaired. Additionally, the inhibition of mitochondrial biogenesis and related genes (PGC-1, SIRT-1, and NRF) occurs. Simultaneously, senescent MSCs in diabetic conditions can further enhance the senescence state by secreting SASPs, which convert normal cells in the surrounding environment into senescent cells. (Prattichizzo et al., 2018; Berlanga-Acosta et al., 2020).

Insulin application may contribute to the senescence of MSCs. Research has shown that insulin can induce senescence in BMSCs by inhibiting autophagy and upregulating the TGF-β1 pathway-related receptor II (TβRII). Furthermore, insulin also impairs the osteogenic differentiation capacity of BMSCs. (Zhang et al., 2020).

### 4.5 The obesity microenvironment

Obesity is a chronic, low-grade inflammatory state that contributes to bone loss and accelerates cellular aging. It is frequently linked to the onset of cardiovascular disease and other metabolic disorders (Li et al., 2020). In the obese microenvironment, MSCs exhibit various signs of early aging. The specific variations in aging-related manifestations of MSCs depend on intra-tissue signaling and the tissue source.

Alessio et al. conducted a study where they isolated MSCs from the body tissues of obese mice and compared them to MSCs derived from normal mice. The findings revealed several differences between the two groups. Firstly, the proliferation rate of MSCs from obese mice was reduced, as indicated by a lower percentage of S-phase cells. Additionally, there was an increased percentage of senescent cells in the obese mice. The expression of senescence-related genes RB21, p21, and p16 was found to be upregulated in MSCs from obese mice. Furthermore, the intracellular ROS levels were increased in these cells. Lastly, the DNA repair capacity of MSCs from obese mice was impaired (Alessio et al., 2020).

The senescence expression was more pronounced in white ADSCs than BMSCs, which could be attributed to the endocrine dysfunction associated with inflammation in white adipose tissue. Li Y et al. (Li et al., 2020) investigated the mechanism behind the altered senescence and differentiation ability of BMSCs caused by obesity. They discovered that by knocking down IL-6, the restoration of osteogenic function was facilitated, the lipogenic tendency of BMSCs was inhibited, and the senescence tendency caused by obesity was suppressed. These effects were potentially linked to the inhibition of the IL-6/STAT3 pathway. In a separate study, Xiang QY’s team (Xiang et al., 2020) explored the impact of postprandial triglyceride-rich lipoproteins (postprandial TRL) on the aging of ADSCs. They observed that the dose size and duration of application of postprandial TRL regulated the aging process of ADSCs. Furthermore, they identified the SIRT1/p53/Ac-p53/p21 pathway as the regulatory pathway for postprandial TRL-induced ADSCs.

However, the effects of obesity on MSCs metabolism and lipogenic differentiation have not been unanimously agreed upon by researchers. Differences in these results may be attributed to the origins of the MSCs. Chen JR et al. (Chen et al., 2016) isolated umbilical cord MSCs from obese pregnant women and found that these cells exhibited impaired lipogenic differentiation and osteogenesis, overexpression of p53, and lower levels of glucose metabolism (glycolysis and oxidative phosphorylation). Tencerova M et al. (Tencerova et al., 2019) discovered that BMSCs from obese patients showed increased expression of lipogenic differentiation genes (PPARG, FASN, IRS1). They also confirmed that activating insulin signaling reduced BMSCs' glycolytic efficiency, increased oxidative phosphorylation, and raised intracellular ROS levels, thus making the cells more prone to senescence.

### 4.6 Microenvironment of hematologic malignancies

The development of leukemic cells in the bone marrow (BM) can negatively impact the survival of MSCs in humans. Studies have shown that BMSCs derived from both acute myeloid leukemia (AML) and chronic myeloid leukemia (CML) sources exhibit senescence-related changes. (Kim et al., 2015; Kumar et al., 2018). Co-culturing MSCs with AML cells leads to a higher preference for oxidative phosphorylation over glycolysis in energy production. Additionally, the altered metabolic patterns observed in the leukemic disease are also linked to the worsening of the disease (Zhang Leisheng et al., 2021; Zhang Luwen et al., 2022). Bonilla X, Vanegas NP, et al. (Bonilla et al., 2019) developed an *in vitro* model called the leukemic ecotone (LN) model. They found that when induced with leukemic cells, MSCs exhibited senescence-related characteristics, such as increased SA-β-gal activity, elevated p53 expression, higher levels of intracellular ROS, and cell cycle arrest. Additionally, these MSCs showed increased secretion of pro-inflammatory factors IL-6, IL-8, and CCL2(Vernot et al., 2017). The researchers then compared LN-MSCs with MSCs derived from patients with B-lymphoblastic acute leukemia (B-ALL). They observed a similar tendency towards senescence, but these changes were reversible. Upon early removal of the leukemia cell effects, B-ALL-derived MSCs reversed senescence and re-entered the cell cycle (Vanegas et al., 2021). The differentiation ability of leukemic MSCs is still a subject of debate, with some researchers suggesting a tendency towards lipogenesis (Le et al., 2016; Azadniv et al., 2020). Furthermore, leukemic MSCs have been found to exhibit increased osteogenic capacity (Battula et al., 2017). The variations in these findings may be attributed to the specific type of leukemia and leukemic cell types involved.

Multiple myeloma (MM) is the second most common malignant hematological disorder, accounting for 13% of all malignant hematological disorders (Kumar et al., 2017). It is characterized by increased osteoclast activity and decreased osteoblast activity. The osteogenic differentiation capacity of bone BMSCs from MM patients is severely impaired in developing the lesion (Arnulf et al., 2007; Corre et al., 2007). The same is true for MM-derived ADSCs: while morphology, proliferation, and lipogenic differentiation capacity are similar to normal ADSCs, the osteogenic differentiation capacity is severely impaired, and SA-β-gal activity is increased. These functional changes may be related to DKK-1 expression (Bereziat et al., 2019).

Kutyna MM’s team (Kutyna et al., 2022)investigated therapy-related myeloid neoplasm (tMN) patient-derived BMSCs and observed aging-related manifestations, including morphological changes, increased expression of aging genes, impaired DNA repair, and reduced lipogenic capacity. Interestingly, the osteogenic differentiation potential of these BMSCs was enhanced, and their energy metabolism tended to shift towards a glycolytic mode compared to normal BMSCs. In normal BMSCs, the mitochondrial OXPHOS: glycolytic ATP production rate was 64%:36%, whereas in tMN-derived BMSCs, it was only 31%:69%. Furthermore, tMN patient-derived BMSCs exhibited a more pronounced trend towards senescence compared to untreated myeloma patient-derived BMSCs, which could be attributed to DNA damage caused by cytotoxic treatment.

When MSCs derived from patients with myelodysplastic syndromes (MDS) are cultured in a laboratory setting, they show signs of senescence. This includes changes in cell shape, with cells becoming enlarged and flattened, as well as a decrease in their ability to divide. Additionally, there is an increase in the levels of a protein called SA-β-gal (Mattiucci et al., 2018), which is associated with high expression of S100A9 in MDS patients. This protein can induce senescence in MSCs through a signaling pathway involving TLR4, NLRP3, and IL-1β secretion (Kim et al., 2012; Shi et al., 2019).

### 4.7 Microenvironment of inborn errors of metabolism

Inborn errors of metabolism are caused by mutations in chromosomal genes, leading to the deletion or abnormality of enzymes. This disruption in the catalytic process of specific enzymes hinders normal metabolic processes. As a result, there is an accumulation of abnormal metabolic substrates and a deficiency of normal products, which affects the organism’s normal development (Agana et al., 2018). Inborn metabolic disorders encompass various types with complex metabolic defects, and a perfect classification method has yet to be established. Previous research has primarily focused on experimental studies of stem cell therapy for inborn metabolic disorders related to the connective tissue, musculoskeletal, neurological, and hematopoietic systems (Ricci and Cacialli, 2021; Specchio et al., 2021). For instance, MSCs and their EVs have shown potential in treating corneal diseases caused by Mucopolysaccharidoses by delivering the enzyme N-acetylgalactosamine-6-sulfate sulfatase (GALNS) to defective cells (Flanagan et al., 2021). Moreover, MSCs and EVs can secrete endogenous coagulation factors FVIII and FIX, making them a promising therapeutic strategy for treating hemophilia (Sokal et al., 2015).

However, senescence-related changes in MSCs derived from the microenvironment of inborn errors of metabolism may have a detrimental impact on their efficacy. For instance, in glycogen storage disease type Ib (GSD-Ib), the ability of MSCs to differentiate into bone and fat cells is hindered due to the absence of glucose-6-phosphate transport protein (G6PT) and the suppression of the OHPOXS response (Sim et al., 2020). The differentially expressed genes (DEGs) in MSCs derived from premature aging syndromes (PAS) compared to MSCs derived from individuals without these syndromes primarily revolve around DNA double-strand damage, telomere damage, and DNA methylation. These genetic changes are associated with the impaired differentiation of MSCs(Trani et al., 2022). In response to these alterations in MSCs, the application of MSC-based cell therapies combined with genetic engineering can provide a safe and effective method for individuals to produce factors that are needed by cells in recipient organs with enzymatic or other defects (Meyerrose et al., 2010) (Tables 1 and 2).

**TABLE 1:** Effects of different microenvironments on the senescence of MSCs.

Microenvironment		Effect	Mechanism	Ref
Aging microenvironment		Cell senescence↑	JAK/STAT &MAPK↑	Peng et al. (2022)
		Cell senescence↑	SASPs; EVs	Ritschka et al., 2017; Sun et al., 2022b
			p21↑	Liu et al., 2017; Chen et al., 2021a
			γH2AX↑	
		Proliferative capacity↓	Cyr61in ECM↓	Marinkovic et al. (2022)
		Differentiation capacity↓	CD137↑	Han et al. (2022)
		Migration ability↓	DPP4, Egf, Actn3, Rho, Cav1↓	Amini-Nik et al. (2022)
Hypoxic/ischaemic microenvironment	Physiological hypoxic	Cell senescence↓	AIMP3↑	Buravkova et al., 2014; Kim et al., 2019
		Cell viability↑	HIF-1α↑	Luo et al. (2019)
			LPL, PKM, & MAP3K13↑	Peck et al. (2021)
		Angiogenic capacity↑	VEGF↑	Kim et al. (2019)
		Osteogenic differentiation↑	RUNX2 & OCN↑	Zhang et al. (2018)
	Pathological ischemic and hypoxic	Cell senescence↑	ROS↑	Rehman et al. (2017)
		Cell viability↓	Baxandcleaved-caspase3↑	Qi et al. (2021)
		Proliferative capacity↓	PI3K/AKT↓	Chen et al. (2022b)
Microenvironment of Immune diseases	SLE	Proliferative capacity↓	LncRNA H19↑	Chen et al. (2021b)
		Immunomodulatory dysfunction	let-7f↓	(Geng et al., 2020)
		Cell apoptosis↑	IL-6↑	
		Cell senescence↑	SASPs↑; JAK-STAT↑; HMGB1↑	Gao and Bird, 2017; Ji et al., 2017
	AS	Osteogenic differentiation↑	Dkk-1↓; RUNX2, OSX,& ALP↑	Daoussis et al. (2022)
		Cell senescence↑	AOPPs↑; ROS↑; p53, p21, & p16↑	Karakoc et al., 2007; Sun et al., 2018
	IBD	Cell senescence↑	p16↑; Paracrine effects	Onyiah and Colgan (2016), Wang et al., 2020
	MS	Cell senescence↑	Telomere loss; SOD1&GSTP↓	Redondo et al., 2018b; Redondo et al., 2018c
	NMO	Proliferative capacity↓ Cell senescence↑	Fas↑; Bcl-xl↓	Yang et al. (2019)
	Pulmonary fibrosis	Proliferative capacity↓ Differentiation capacity↓ Cell senescence↑	NADH-AMPK-p53↑	Cardenes et al. (2018)
Hyperglycemic microenvironment		Cell senescence↑	p16, p21, &p27↑; p53↑ Metabolic alteration (Oxidative phosphorylation↑; ROS↑; DNA damage↑	Kong et al., 2019; Yin et al., 2021a; Wu, 2021
		Osteogenic and chondrogenic differentiation ↓		Kong et al. (2019)
Obesity microenvironment		Proliferative capacity↓	RB21, p21, & p16↑; ROS↑; DNAdamage↑	Alessio et al. (2020)
		Cell senescence↑		
		Differentiation capacity↓	p53↑; Glucose metabolism↓	Chen et al. (2016)
		Lipogenic differentiation↑	PARG, FASN, IRS1↑	Tencerova et al. (2019)
Microenvironment of hematologic malignancies	AML&CML	Cell viability↓ Cell senescence↑	Oxidative Phosphorylation↑	Kim et al., 2015; Kumar et al., 2018
	LN	Cell senescence↑	p53↑; ROS↑; IL-6, IL-8, && CCL2↑	Vernot et al. (2017)
	MM	Osteogenic differentiation↓	DKK-1↑	Bereziat et al. (2019)
	tMN	Cell senescence↑ Lipogenic differentiation↓ Osteogenic differentiation↑ Metabolic alteration		Kutyna et al. (2022)
	MDS	Cell senescence↑ Proliferation capacity↓	S100A9↑; TLR4-NLRP3-IL-1β↑	Kim et al., 2012; Shi et al., 2019

**TABLE 2:** Typical clinical trials of MSCs.

Condition/Disease	NCT number	Study title	Phase	Sponsor
Healthy	NCT04313647	A Tolerance Clinical Study on Aerosol Inhalation of Mesenchymal Stem Cells Exosomes In Healthy Volunteers	Phase 1	Ruijin Hospital
	NCT01087996	The Percutaneous Stem Cell Injection Delivery Effects on Neomyogenesis Pilot Study (The POSEIDON-Pilot Study)	Phase1	University of Miami
			Phase 2	
Aging Frailty	NCT02065245	AllogeneiC Human Mesenchymal Stem Cells (hMSC) in Patients With Aging FRAilTy Via IntravenoUS Delivery	Phase1	Longeveron Inc
			Phase 2	
COVID-19	NCT04349631	A Clinical Trial to Determine the Safety and Efficacy of HB-adMSCs to Provide Protection Against COVID-19	Phase 2	Hope Biosciences Stem Cell Research Foundation
	NCT04362189	Efficacy and Safety Study of Allogeneic HB-adMSCs for the Treatment of COVID-19	Phase 2	Hope Biosciences Stem Cell Research Foundation
	NCT04348435	A Randomized, Double-Blind, Single Center, Efficacy and Safety Study of Allogeneic HB-adMSCs Against COVID-19	Phase 2	Hope Biosciences Stem Cell Research Foundation
	NCT04399889	hCT-MSCs for COVID-19 ARDS	Phase1	Joanne Kurtzberg, MD
			Phase 2	
	NCT04493242	Extracellular Vesicle Infusion Treatment for COVID-19 Associated ARDS	Phase 2	Direct Biologics, LLC
Acute Respiratory Distress Syndrome	NCT01775774	Human Mesenchymal Stem Cells For Acute Respiratory Distress Syndrome	Phase 1	Michael A. Matthay
	NCT04355728	Use of UC-MSCs for COVID-19 Patients	Phase1	Camillo Ricordi
			Phase 2	
Idiopathic Pulmonary Fibrosis	NCT01385644	A Study to Evaluate the Potential Role of Mesenchymal Stem Cells in the Treatment of Idiopathic Pulmonary Fibrosis	Phase 1	The Prince Charles Hospital
Bronchopulmonary Dysplasia	NCT03857841	A Safety Study of IV Stem Cell-derived Extracellular Vesicles (UNEX-42) in Preterm Neonates at High Risk for BPD	Phase 1	United Therapeutics
Ischemic Heart Failure	NCT02501811	Combination of Mesenchymal and C-kit + Cardiac Stem Cells as Regenerative Therapy for Heart Failure	Phase 2	The University of Texas Health Science Center, Houston
	NCT03925324	Serial Infusions of Allogeneic Mesenchymal Stem Cells in Cardiomyopathy Patients With Left Ventricular Assist Device	Phase 2	Medstar Health Research Institute
	NCT00768066	The Transendocardial Autologous Cells (hMSC or hBMC) in Ischemic Heart Failure Trial (TAC-HFT)	Phase1	University of Miami
			Phase 2	
	NCT00587990	Prospective Randomized Study of Mesenchymal Stem Cell Therapy in Patients Undergoing Cardiac Surgery (PROMETHEUS)	Phase1	Joshua M Hare
			Phase 2	
	NCT02013674	The Transendocardial Stem Cell Injection Delivery Effects on Neomyogenesis STudy (The TRIDENT Study)	Phase 2	Joshua M Hare
	NCT01781390	Safety Study of Allogeneic Mesenchymal Precursor Cell Infusion in Myocardial Infarction	Phase 2	Mesoblast, Inc
	NCT01270139	Plasmonic Nanophotothermal Therapy of Atherosclerosis	Not Applicable	Ural State Medical University
	NCT00927784	Effect of Intramyocardial Injection of Mesenchymal Precursor Cells on Heart Function in People Receiving an LVAD	Phase 2	Icahn School of Medicine at Mount Sinai
Non-ischemic Heart Failure	NCT01392625	PercutaneOus StEm Cell Injection Delivery Effects On Neomyogenesis in Dilated CardioMyopathy (The POSEIDON-DCM Study)	Phase1	Joshua M Hare
			Phase 2	
	NCT02467387	A Study to Assess the Effect of Intravenous Dose of (aMBMC) to Subjects With Non-ischemic Heart Failure	Phase 2	CardioCell LLC
Cairdiomyopathy Due to Anthracyclines	NCT02509156	Stem Cell Injection in Cancer Survivors	Phase1	The University of Texas Health Science Center, Houston
Tendon Injury	NCT02298023	Treatment of Tendon Injury Using Allogenic Adipose-derived Mesenchymal Stem Cells (Rotator Cuff Tear)	Phase 2	Seoul National University Hospital
Focal articular cartilage lesions of the knee	NCT02037204	IMPACT: Safety and Feasibility of a Single-stage Procedure for Focal Cartilage Lesions of the Knee	Phase1	UMC Utrecht
			Phase 2	
Spinal Cord Injury	NCT02481440	Repeated Subarachnoid Administrations of hUC-MSCs in Treating SCI	Phase1	Limin Rong
			Phase 2	
	NCT01909154	Safety Study of Local Administration of Autologous Bone Marrow Stromal Cells in Chronic Paraplegia	Phase 1	Puerta de Hierro University Hospital
Osteoarthritis	NCT01586312	Treatment of Knee Osteoarthritis With Allogenic Mesenchymal Stem Cells	Phase1	Red de Terapia Celular
			Phase 2	
	NCT02958267	Investigation of Mesenchymal Stem Cell Therapy for the Treatment of Osteoarthritis of the Knee	Phase 2	OhioHealth
	NCT01183728	Treatment of Knee Osteoarthritis With Autologous Mesenchymal Stem Cells	Phase1	Red de Terapia Celular
			Phase 2	
	NCT02674399	A Phase 2 Study to Evaluate the Efficacy and Safety of JointStem in Treatment of Osteoarthritis	Phase 2	Nature Cell Co. Ltd
Rheumatoid Arthritis	NCT03691909	Phase 1/2a Clinical Trial to Assess the Safety of HB-adMSCs for the Treatment of Rheumatoid Arthritis	Phase1	Hope Biosciences
			Phase 2	
Degeneration Articular Cartilage Knee	NCT01733186	Evaluation of Safety and Exploratory Efficacy of CARTISTEM? a Cell Therapy Product for Articular Cartilage Defects	Phase1	Medipost Co. Ltd
			Phase 2	
Multiple Sclerosis, Chronic Progressive	NCT03799718	Safety and Efficacy of Repeated Administration of NurOwn (MSC-NTF Cells) in Participants With Progressive MS	Phase 2	Brainstorm-Cell Therapeutics
Xerostomia	NCT02513238	Mesenchymal Stemcells for Radiation Induced Xerostomia	Phase 2	Rigshospitalet, Denmark
Cystic Fibrosis	NCT02866721	Safety and Tolerability Study of Allogeneic Mesenchymal Stem Cell Infusion in Adults With Cystic Fibrosis	Phase1	University Hospitals Cleveland Medical Center
Acute Graft Versus Host Disease	NCT02379442	Early Treatment of Acute Graft Versus Host Disease With Bone Marrow-Derived Mesenchymal Stem Cells and Corticosteroids	Phase1	National Heart, Lung, and Blood Institute (NHLBI)
			Phase 2	
Diabetes Mellitus	NCT02886884	Allogeneic Mesenchymal Human Stem Cells Infusion Therapy for Endothelial DySfunctiOn in Diabetic Subjects	Phase1	Joshua M Hare
			Phase 2	
	NCT02387749	Effect Of Mesenchymal Stem Cells Transfusion on the Diabetic Peripheral Neuropathy Patients	Not Applicable	Cairo University
Metabolic Syndrome	NCT03059355	Infusion of Umbilical Cord Versus Bone Marrow Derived Mesenchymal Stem Cells to Evaluate Cytokine Suppression	Phase1	Joshua M Hare
			Phase 2	
Breast Reconstruction	NCT01771913	Immunophenotyping of Fresh Stromal Vascular Fraction From Adipose Derived Stem Cells (ADSC) Enriched Fat Grafts	Phase 2	University of Sao Paulo
Alzheimer Disease	NCT03117738	A Study to Evaluate the Safety and Efficacy of AstroStem in Treatment of Alzheimer’s Disease	Phase1	Nature Cell Co. Ltd
			Phase 2	
Cerebral Palsy	NCT03473301	A Study of UCB and MSCs in Children With CP: ACCeNT-CP	Phase1	Joanne Kurtzberg, MD
			Phase 2	
Cleft Lip and Palate	NCT01932164	Use of Mesenchymal Stem Cells for Alveolar Bone Tissue Engineering for Cleft Lip and Palate Patients	Not Applicable	Hospital Sirio-Libanes
Dental Pulp Regeneration	NCT03102879	Encapsulated Mesenchymal Stem Cells for Dental Pulp Regeneration	Not Applicable	Universidad de los Andes, Chile
Retinal Degeneration	NCT02330978	Intravitreal Mesenchymal Stem Cell Transplantation in Advanced Glaucoma	Phase 1	University of Sao Paulo
Malignant Melanoma	NCT02331134	Tissue and Hematopoietic/Mesenchymal Stem Cell for Humanized Xenograft Studies in Melanoma and Squamous Head and Neck Cancer	Not Applicable	University of Colorado, Denver

## 5 Conclusion

MSCs have gained significant attention in the fields of organ repair, new drug development, anti-aging, and rare disease treatment due to their ability to differentiate in multiple directions and their immunological properties. However, along with these promising possibilities, there are also emerging challenges. These include establishing quality standards for MSCs, ensuring their activity during *in vitro* culture, guaranteeing the survival and functionality of MSCs at the site of the disease to achieve therapeutic effects, understanding the potential negative effects of drugs used to treat the primary pathology on MSCs, and investigating the role of MSCs in tumorigenesis (Nowak et al., 2021). In this review, we provide a summary of the effects and mechanisms of different microenvironments on the senescence and function of MSCs. We also discuss possible ways to improve and explore further research directions. These include: 1. Pre-treating MSCs with specific media before transplantation or culture to enhance their survival rate post-transplantation. 2. Selecting the most suitable MSC donors to ensure efficient utilization of MSCs. 3. Exploring the combination of drugs, bioactive signals, natural and synthetic materials (such as Hydrogels and scaffolds) with MSCs. 4. Investigating the utilization of EVs as an alternative to MSCs and modifying them to address ethical concerns and potential carcinogenic effects. Understanding the interaction between MSCs and the cellular microenvironment is crucial for advancing MSC therapeutics and fostering realistic possibilities for their clinical application. Up to now, MSCs have been the subject of more than 1,599 clinical trials investigating their potential for treatment, with most of these trials still in the early stages. Although the preliminary data from these trials are promising, only two of them involve pretreatment (NCT03105284 and NCT01962233), indicating a lack of rigorous and uniform effective means of establishing a culture system related to pretreatment. Furthermore, MSC therapy still lacks long-term safety assessment, and large-scale and controlled trials are needed to make more conclusive judgments about MSC-based therapies, which are important for clinical translation. It is worth noting that clinical patients often suffer from multiple diseases, not a single disease, and most animal experiments with MSCs have focused on only a single disease model. Therefore, exploring the efficacy of MSCs under multiple diseases in future clinical translation is one of the future research priorities.

## References

[B1] Acosta J. C. Banito A. Wuestefeld T. Georgilis A. Janich P. Morton J. P. (2013). A complex secretory program orchestrated by the inflammasome controls paracrine senescence. Nat. Cell. Biol. 15 (8), 978–990. 10.1038/ncb2784 23770676 PMC3732483

[B2] Acosta J. C. O'Loghlen A. Banito A. Guijarro M. V. Augert A. Raguz S. (2008). Chemokine signaling via the CXCR2 receptor reinforces senescence. Cell. 133 (6), 1006–1018. 10.1016/j.cell.2008.03.038 18555777

[B3] Agana M. Frueh J. Kamboj M. Patel D. R. Kanungo S. (2018). Common metabolic disorder (inborn errors of metabolism) concerns in primary care practice. Ann. Transl. Med. 6 (24), 469. 10.21037/atm.2018.12.34 30740400 PMC6331353

[B4] Ahmadi M. Rezaie J. (2021). Ageing and mesenchymal stem cells derived exosomes: molecular insight and challenges. Cell. Biochem. Funct. 39 (1), 60–66. 10.1002/cbf.3602 33164248

[B5] Al Aboud N. M. Tupper C. Jialal I. (2023). “Genetics, epigenetic mechanism,” in *StatPearls*. Treasure Island (FL) ineligible companies. Disclosure: connor Tupper declares no relevant financial relationships with ineligible companies. Disclosure: ishwarlal Jialal declares no relevant financial relationships with ineligible companies (United States: StatPearls Publishing).

[B6] Al-Azab M. Safi M. Idiiatullina E. Al-Shaebi F. MohamedZaky Y. (2022). Aging of mesenchymal stem cell: machinery, markers, and strategies of fighting. Cell. Mol. Biol. Lett. 27 (1), 69. 10.1186/s11658-022-00366-0 35986247 PMC9388978

[B7] Al-Azab M. Wang B. Elkhider A. Williams W. Li W. Yuan Bo (2020). Indian Hedgehog regulates senescence in bone marrow-derived mesenchymal stem cell through modulation of ROS/mTOR/4EBP1, p70S6K1/2 pathway. Aging-Us 12 (7), 5693–5715. 10.18632/aging.102958 PMC7185126 32235006

[B8] Alessio N. Acar M. B. Demirsoy I. H. Squillaro T. Siniscalco D. Di Bernardo G. (2020). Obesity is associated with senescence of mesenchymal stromal cells derived from bone marrow, subcutaneous and visceral fat of young mice. Aging-Us 12 (13), 12609–12621. 10.18632/aging.103606 PMC7377882 32634118

[B9] Almeida M. Porter R. M. (2019). Sirtuins and FoxOs in osteoporosis and osteoarthritis. Bone 121, 284–292. 10.1016/j.bone.2019.01.018 30738214 PMC6812652

[B10] Alpdundar Bulut E. Bayyurt Kocabas B. Yazar V. Aykut G. Guler U. Salih B. (2020). Human gut commensal membrane vesicles modulate inflammation by generating M2-like macrophages and myeloid-derived suppressor cells. J. Immunol. 205 (10), 2707–2718. 10.4049/jimmunol.2000731 33028617

[B11] Al Suraih M. S. Trussoni C. E. Splinter P. L. LaRusso N. F. O'Hara S. P. (2020). Senescent cholangiocytes release extracellular vesicles that alter target cell phenotype via the epidermal growth factor receptor. Liver Int. 40 (10), 2455–2468. 10.1111/liv.14569 32558183 PMC7669612

[B12] Amini-Nik S. Abdullahi A. Vinaik R. Ren J. Yao R. Yu N. (2022). Aging impairs the cellular interplay between myeloid cells and mesenchymal cells during skin healing in mice. Aging Dis. 13 (2), 540–551. 10.14336/ad.2021.1008 35371611 PMC8947831

[B13] Anastasiadou E. Ceccarelli S. Messina E. Gerini G. Megiorni F. Pontecorvi P. (2021). MiR-200c-3p maintains stemness and proliferative potential in adipose-derived stem cells by counteracting senescence mechanisms. Plos One 16 (9), e0257070. 10.1371/journal.pone.0257070 34534238 PMC8448302

[B14] Antonioli E. Torres N. Ferretti M. Piccinato C. A. Sertie A. L. (2019). Individual response to mTOR inhibition in delaying replicative senescence of mesenchymal stromal cells. PLoS One 14 (1), e0204784. 10.1371/journal.pone.0204784 30703123 PMC6354956

[B15] Arnulf B. Lecourt S. Soulier J. Ternaux B. Noelle Lacassagne M. Crinquette A. (2007). Phenotypic and functional characterization of bone marrow mesenchymal stem cells derived from patients with multiple myeloma. Leukemia 21 (1), 158–163. 10.1038/sj.leu.2404466 17096013

[B16] Azadniv M. Myers J. R. McMurray H. R. Guo N. Rock P. Coppage M. L. (2020). Bone marrow mesenchymal stromal cells from acute myelogenous leukemia patients demonstrate adipogenic differentiation propensity with implications for leukemia cell support. Leukemia 34 (2), 391–403. 10.1038/s41375-019-0568-8 31492897 PMC7214245

[B17] Bai M. Zhang Li Fu Bo Bai J. Zhang Y. Cai G. (2018). IL-17A improves the efficacy of mesenchymal stem cells in ischemic-reperfusion renal injury by increasing Treg percentages by the COX-2/PGE2 pathway. Kidney Int. 93 (4), 814–825. 10.1016/j.kint.2017.08.030 29132705

[B18] Banimohamad-Shotorbani B. Kahroba H. Sadeghzadeh H. Wilson D. M. 3rd Maadi H. Samadi N. (2020). DNA damage repair response in mesenchymal stromal cells: from cellular senescence and aging to apoptosis and differentiation ability. Ageing Res. Rev. 62, 101125. 10.1016/j.arr.2020.101125 32683038

[B19] Basisty N. Kale A. Jeon O. H. Kuehnemann C. Payne T. Rao C. (2020). A proteomic atlas of senescence-associated secretomes for aging biomarker development. PLoS Biol. 18 (1), e3000599. 10.1371/journal.pbio.3000599 31945054 PMC6964821

[B20] Battula V. L. Le P. M. Sun J. C. Nguyen K. Yuan B. Zhou X. (2017). AML-induced osteogenic differentiation in mesenchymal stromal cells supports leukemia growth. Jci Insight 2 (13), e90036. 10.1172/jci.insight.90036 28679949 PMC5499365

[B21] Bereziat V. Mazurier C. Auclair M. Ferrand N. Jolly S. Marie T. (2019). Systemic dysfunction of osteoblast differentiation in adipose-derived stem cells from patients with multiple myeloma. Cells 8 (5), 441. 10.3390/cells8050441 31083455 PMC6562713

[B22] Berlanga-Acosta J. A. Guillen-Nieto G. E. Rodriguez-Rodriguez N. Mendoza-Mari Y. Luisa Bringas-Vega M. JorgeBerlanga-Saez O. (2020). Cellular senescence as the pathogenic hub of diabetes-related wound chronicity. Front. Endocrinol. 11, 573032. 10.3389/fendo.2020.573032 PMC7525211 33042026

[B23] Bernadotte A. Mikhelson V. M. Spivak I. M. (2016). Markers of cellular senescence. Telomere shortening as a marker of cellular senescence. Aging (Albany NY) 8 (1), 3–11. 10.18632/aging.100871 26805432 PMC4761709

[B24] Bernard M. Yang B. Migneault F. Turgeon J. Dieude M. Olivier M. A. (2020). Autophagy drives fibroblast senescence through MTORC2 regulation. Autophagy 16 (11), 2004–2016. 10.1080/15548627.2020.1713640 31931659 PMC7595590

[B25] Blackburn E. H. Gall J. G. (1978). A tandemly repeated sequence at the termini of the extrachromosomal ribosomal RNA genes in Tetrahymena. J. Mol. Biol. 120 (1), 33–53. 10.1016/0022-2836(78)90294-2 642006

[B26] Block T. J. Marinkovic M. Tran O. N. Amanda Marshall A. O. G. Dean D. D. (2017). Restoring the quantity and quality of elderly human mesenchymal stem cells for autologous cell-based therapies. Stem Cell. Res. Ther. 8, 239. 10.1186/s13287-017-0688-x 29078802 PMC5658952

[B27] Bonab M. M. Alimoghaddam K. Talebian F. Ghaffari S. H. Ghavamzadeh A. Nikbin B. (2006). Aging of mesenchymal stem cell *in vitro* . BMC Cell. Biol. 7, 14. 10.1186/1471-2121-7-14 16529651 PMC1435883

[B28] Bonilla X. Vanegas N.-D. P. Paul Vernot J. (2019). Acute leukemia induces senescence and impaired osteogenic differentiation in mesenchymal stem cells endowing leukemic cells with functional advantages. Stem Cells Int. 2019, 3864948. 10.1155/2019/3864948 31065273 PMC6466857

[B29] Bork S. Pfister S. Witt H. Horn P. Korn B. Ho A. D. (2010). DNA methylation pattern changes upon long-term culture and aging of human mesenchymal stromal cells. Aging Cell. 9 (1), 54–63. 10.1111/j.1474-9726.2009.00535.x 19895632 PMC2814091

[B30] Briscoe J. Thérond P. P. (2013). The mechanisms of Hedgehog signalling and its roles in development and disease. Nat. Rev. Mol. Cell. Biol. 14 (7), 416–429. 10.1038/nrm3598 23719536

[B31] Brunet A. Goodell M. A. Rando T. A. (2023). Ageing and rejuvenation of tissue stem cells and their niches. Nat. Rev. Mol. Cell. Biol. 24 (1), 45–62. 10.1038/s41580-022-00510-w 35859206 PMC9879573

[B32] Buravkova L. B. Andreeva E. R. Gogvadze V. Zhivotovsky B. (2014). Mesenchymal stem cells and hypoxia: where are we? Mitochondrion 19, 105–112. 10.1016/j.mito.2014.07.005 25034305

[B33] Cakouros D. Gronthos S. (2020). The changing epigenetic landscape of Mesenchymal Stem/Stromal Cells during aging. Bone 137, 115440. 10.1016/j.bone.2020.115440 32445894

[B34] Canli O. Nicolas A. M. Gupta J. Finkelmeier F. Goncharova O. Pesic M. (2017). Myeloid cell-derived reactive oxygen species induce epithelial mutagenesis. Cancer Cell. 32 (6), 869–883. 10.1016/j.ccell.2017.11.004 29232557

[B35] Cardenes N. Alvarez D. Sellares J. Peng Y. Corey C. Wecht S. (2018). Senescence of bone marrow-derived mesenchymal stem cells from patients with idiopathic pulmonary fibrosis. Stem Cell. Res. Ther. 9, 257. 10.1186/s13287-018-0970-6 30257725 PMC6158816

[B36] Chaib S. Tchkonia T. Kirkland J. L. (2022). Cellular senescence and senolytics: the path to the clinic. Nat. Med. 28 (8), 1556–1568. 10.1038/s41591-022-01923-y 35953721 PMC9599677

[B37] Chang J. T. (2020). Pathophysiology of inflammatory bowel diseases. N. Engl. J. Med. 383 (27), 2652–2664. 10.1056/NEJMra2002697 33382932

[B38] Chapman J. Fielder E. Passos J. F. (2019). Mitochondrial dysfunction and cell senescence: deciphering a complex relationship. Febs Lett. 593 (13), 1566–1579. 10.1002/1873-3468.13498 31211858

[B39] Chen C. Xia S. He J. Lu G. Xie Z. Han H. (2019). Roles of taurine in cognitive function of physiology, pathologies and toxication. Life Sci. 231, 116584. 10.1016/j.lfs.2019.116584 31220527

[B40] Chen C. Zhou M. Ge Y. Wang X. (2020a). SIRT1 and aging related signaling pathways. Mech. Ageing Dev. 187, 111215. 10.1016/j.mad.2020.111215 32084459

[B41] Chen H. Liu O. Chen S. Zhou Y. (2022a). Aging and mesenchymal stem cells: therapeutic opportunities and challenges in the older group. Gerontology 68 (3), 339–352. 10.1159/000516668 34161948 PMC8985028

[B42] Chen J. (2016). The cell-cycle arrest and apoptotic functions of p53 in tumor initiation and progression. Cold Spring Harb. Perspect. Med. 6 (3), a026104. 10.1101/cshperspect.a026104 26931810 PMC4772082

[B43] Chen J. R. Lazarenko O. P. Blackburn M. L. Rose S. Frye R. E. Badger T. M. (2016). Maternal obesity programs senescence signaling and glucose metabolism in osteo-progenitors from rat and human. Endocrinology 157 (11), 4172–4183. 10.1210/en.2016-1408 27653035

[B44] Chen W. Baylink D. J. Brier-Jones J. Neises A. Kiroyan J. B. Rundle C. H. (2015). PDGFB-based stem cell gene therapy increases bone strength in the mouse. Proc. Natl. Acad. Sci. U. S. A. 112 (29), E3893–E3900. 10.1073/pnas.1501759112 26150503 PMC4517286

[B45] Chen X. Feng J. Chang Q. Lu F. Yuan Yi (2021a). Senescence of donor cells impairs fat graft regeneration by suppressing adipogenesis and increasing expression of senescence-associated secretory phenotype factors. Stem Cell. Res. Ther. 12 (1), 311. 10.1186/s13287-021-02383-w 34051860 PMC8164816

[B46] Chen X. Luo X. Wei Y. Sun H. Dai L. Tangzhou Y. (2021b). LncRNA H19 induces immune dysregulation of BMMSCs, at least partly, by inhibiting IL-2 production. Mol. Med. 27 (1), 61. 10.1186/s10020-021-00326-y 34130625 PMC8207721

[B47] Chen Xi Wang C. Jiang Y. Wang Qi Yu T. Zhang H. (2020b). Bcl-3 promotes Wnt signaling by maintaining the acetylation of beta-catenin at lysine 49 in colorectal cancer. Signal Transduct. Target. Ther. 5 (1), 52. 10.1038/s41392-020-0138-6 32355204 PMC7193563

[B48] Chen X. Zhou C. Xu D. Liu X. Li S. Hou J. (2022b). Peptide hormone ELABELA promotes rat bone marrow-derived mesenchymal stem cell proliferation and migration by manipulating the cell cycle through the PI3K/AKT pathway under the hypoxia and ischemia microenvironment. Stem Cell. Res. Ther. 13 (1), 32. 10.1186/s13287-021-02691-1 35090551 PMC8796437

[B49] Choi J. Hwang M. P. Lee J. W. Lee K. H. (2014). A glimpse into the interactions of cells in a microenvironment: the modulation of T cells by mesenchymal stem cells. Int. J. Nanomedicine 9 (1), 127–139. 10.2147/ijn.S50767 PMC4024981 24872708

[B50] Chondrogianni N. Tzavelas C. Pemberton A. J. Nezis I. P. Rivett A. J. Gonos E. S. (2005). Overexpression of proteasome beta5 assembled subunit increases the amount of proteasome and confers ameliorated response to oxidative stress and higher survival rates. J. Biol. Chem. 280 (12), 11840–11850. 10.1074/jbc.M413007200 15661736

[B51] Chung H. Y. Lee E. K. Choi Y. J. Kim J. M. Kim D. H. Zou Y. (2011). Molecular inflammation as an underlying mechanism of the aging process and age-related diseases. J. Dent. Res. 90 (7), 830–840. 10.1177/0022034510387794 21447699

[B52] Coppe J.-P. Patil C. K. Rodier F. Sun Yu Munoz D. P. Goldstein J. (2008). Senescence-associated secretory phenotypes reveal cell-nonautonomous functions of oncogenic RAS and the p53 tumor suppressor. Plos Biol. 6 (12), 2853–2868. 10.1371/journal.pbio.0060301 19053174 PMC2592359

[B53] Corre J. Mahtouk K. Attal M. Gadelorge M. Huynh A. Fleury-Cappellesso S. (2007). Bone marrow mesenchymal stem cells are abnormal in multiple myeloma. Leukemia 21 (5), 1079–1088. 10.1038/sj.leu.2404621 17344918 PMC2346535

[B54] Czosseck A. Chen M. M. Nguyen H. Meeson A. Hsu C.-C. Chen C.-C. (2022). Porous scaffold for mesenchymal cell encapsulation and exosome-based therapy of ischemic diseases. J. Control. release official J. Control. Release Soc. 352, 879–892. 10.1016/j.jconrel.2022.10.057 36370875

[B55] Damasceno P. K. F. de Santana T. A. Santos G. C. Orge I. D. Silva D. N. Albuquerque J. F. (2020). Genetic engineering as a strategy to improve the therapeutic efficacy of mesenchymal stem/stromal cells in regenerative medicine. Front. Cell. Dev. Biol. 8, 737. 10.3389/fcell.2020.00737 32974331 PMC7471932

[B56] Daoussis D. Kanellou A. Panagiotopoulos E. Papachristou D. (2022). DKK-1 is underexpressed in mesenchymal stem cells from patients with ankylosing spondylitis and further downregulated by IL-17. Int. J. Mol. Sci. 23 (12), 6660. 10.3390/ijms23126660 35743102 PMC9224314

[B57] Davis C. Dukes A. Drewry M. Helwa I. Johnson M. H. Isales C. M. (2017). MicroRNA-183-5p increases with age in bone-derived extracellular vesicles, suppresses bone marrow stromal (stem) cell proliferation, and induces stem cell senescence. Tissue Eng. Part A 23 (21-22), 1231–1240. 10.1089/ten.TEA.2016.0525 28363268 PMC5689127

[B58] Deng J. Q. Ouyang P. Li W. Y. Zhong L. J. Gu C. W. Shen L. H. (2021). Curcumin alleviates the senescence of canine bone marrow mesenchymal stem cells during *in vitro* expansion by activating the autophagy pathway. Int. J. Mol. Sci. 22 (21), 11356. 10.3390/ijms222111356 34768788 PMC8583405

[B59] Di Mitri D. Alimonti A. (2016). Non-cell-autonomous regulation of cellular senescence in cancer. Trends Cell. Biol. 26 (3), 215–226. 10.1016/j.tcb.2015.10.005 26564316

[B60] Dimri G. P. Lee X. Basile G. Acosta M. Scott G. Roskelley C. (1995). A biomarker that identifies senescent human cells in culture and in aging skin *in vivo* . Proc. Natl. Acad. Sci. U. S. A. 92 (20), 9363–9367. 10.1073/pnas.92.20.9363 7568133 PMC40985

[B61] Dominici M. Le Blanc K. Mueller I. Slaper-Cortenbach I. Marini F. C. Krause D. S. (2006). Minimal criteria for defining multipotent mesenchymal stromal cells. The International Society for Cellular Therapy position statement. Cytotherapy 8 (4), 315–317. 10.1080/14653240600855905 16923606

[B62] Engeland K. (2022). Cell cycle regulation: p53-p21-RB signaling. Cell. Death Differ. 29 (5), 946–960. 10.1038/s41418-022-00988-z 35361964 PMC9090780

[B63] Estrada J. C. Torres Y. Benguría A. Dopazo A. Roche E. Carrera-Quintanar L. (2013). Human mesenchymal stem cell-replicative senescence and oxidative stress are closely linked to aneuploidy. Cell. Death Dis. 4 (6), e691. 10.1038/cddis.2013.211 23807220 PMC3702285

[B64] Fan P. Xiao-Yu Yu Xie X.-H. Chen C.-H. Zhang Po Cheng Y. (2019). Mitophagy is a protective response against oxidative damage in bone marrow mesenchymal stem cells. Life Sci. 229, 36–45. 10.1016/j.lfs.2019.05.027 31085242

[B65] Fane M. Weeraratna A. T. (2020). How the ageing microenvironment influences tumour progression. Nat. Rev. Cancer 20 (2), 89–106. 10.1038/s41568-019-0222-9 31836838 PMC7377404

[B66] Flanagan M. Pathak I. Gan Q. Winter L. Emnet R. Akel S. (2021). Umbilical mesenchymal stem cell-derived extracellular vesicles as enzyme delivery vehicle to treat Morquio A fibroblasts. Stem Cell. Res. Ther. 12 (1), 276. 10.1186/s13287-021-02355-0 33957983 PMC8101245

[B67] Friedenstein A. J. Petrakova K. V. Kurolesova A. I. Frolova G. P. (1968). Heterotopic of bone marrow. Analysis of precursor cells for osteogenic and hematopoietic tissues. United States: Transplantation. 5654088

[B68] Gao L. Bird A. K. Meednu N. Dauenhauer K. Liesveld J. Anolik J. (2017). Bone marrow-derived mesenchymal stem cells from patients with systemic lupus erythematosus have a senescence-associated secretory phenotype mediated by a mitochondrial antiviral signaling protein-interferon-beta feedback loop. Arthritis & Rheumatology 69 (8), 1623–1635. 10.1002/art.40142 28471483 PMC5560120

[B69] Garcia-Olmo D. Gilaberte I. Binek M. Hoore Ajl D. Lindner D. Selvaggi F. (2022). Follow-up study to evaluate the long-term safety and efficacy of darvadstrocel (mesenchymal stem cell treatment) in patients with perianal fistulizing Crohn's disease: ADMIRE-CD phase 3 randomized controlled trial. Dis. Colon Rectum 65 (5), 713–720. 10.1097/dcr.0000000000002325 34890373 PMC8985696

[B70] Geng L. Tang X. Wang S. Sun Y. Wang D. Tsao B. P. (2020). Reduced let-7f in bone marrow-derived mesenchymal stem cells triggers Treg/Th17 imbalance in patients with systemic lupus erythematosus. Front. Immunol. 11, 233. 10.3389/fimmu.2020.00233 32133007 PMC7040072

[B71] Greif D. N. Kouroupis D. Murdock C. J. Griswold A. J. Kaplan L. D. Best T. M. (2020). Infrapatellar fat pad/synovium complex in early-stage knee osteoarthritis: potential new target and source of therapeutic mesenchymal stem/stromal cells. Front. Bioeng. Biotechnol. 8, 860. 10.3389/fbioe.2020.00860 32850724 PMC7399076

[B72] Grim C. Noble R. Uribe G. Khanipov K. Johnson P. Koltun W. A. (2021). Impairment of tissue-resident mesenchymal stem cells in chronic ulcerative colitis and Crohn's disease. J. Crohns Colitis 15 (8), 1362–1375. 10.1093/ecco-jcc/jjab001 33506258 PMC8328298

[B73] Guerrero E. N. Vega S. Fu C. De Leon R. Beltran D. Solis M. A. (2021). Increased proliferation and differentiation capacity of placenta-derived mesenchymal stem cells from women of median maternal age correlates with telomere shortening. Aging-Us 13 (22), 24542–24559. 10.18632/aging.203724 PMC8660609 34845112

[B74] Hafner A. Bulyk M. L. Jambhekar A. Lahav G. (2019). The multiple mechanisms that regulate p53 activity and cell fate. Nat. Rev. Mol. Cell. Biol. 20 (4), 199–210. 10.1038/s41580-019-0110-x 30824861

[B75] Han J. Wang Y. Zhou H. Zhang Y. Wan D. (2022). CD137 regulates bone loss *via* the p53 wnt/β-catenin signaling pathways in aged mice. Front. Endocrinol. 13, 922501. 10.3389/fendo.2022.922501 PMC9279613 35846320

[B76] Harrell C. R. Djonov V. Volarevic V. (2022). Therapeutic potential of mesenchymal stem cells in the treatment of ocular graft-versus-host disease. Int. J. Mol. Sci. 23 (21), 13254. 10.3390/ijms232113254 36362040 PMC9656879

[B77] Hayflick L. Moorhead P. S. (1961). The serial cultivation of human diploid cell strains. Exp. Cell. Res. 25, 585–621. 10.1016/0014-4827(61)90192-6 13905658

[B78] He L. Liao J. Liu Z. Wang T. Zhou Y. Wang T. (2023). Multi-omic analysis of mandibuloacral dysplasia type A patient iPSC-derived MSC senescence reveals miR-311 as a novel biomarker for MSC senescence. Hum. Mol. Genet. 32 (19), 2872–2886. 10.1093/hmg/ddad111 37427980

[B79] Hernandez-Segura A. Nehme J. Demaria M. (2018). Hallmarks of cellular senescence. Trends Cell. Biol. 28 (6), 436–453. 10.1016/j.tcb.2018.02.001 29477613

[B80] Herranz N. Gil J. (2018). Mechanisms and functions of cellular senescence. J. Clin. Investig. 128 (4), 1238–1246. 10.1172/jci95148 29608137 PMC5873888

[B81] Hong Y. He H. Jiang G. Zhang H. Tao W. Ding Y. (2020). miR-155-5p inhibition rejuvenates aged mesenchymal stem cells and enhances cardioprotection following infarction. Aging Cell. 19 (4), e13128. 10.1111/acel.13128 32196916 PMC7189985

[B82] Huang J. Zhao L. Xing L. Chen Di (2010). MicroRNA-204 regulates Runx2 protein expression and mesenchymal progenitor cell differentiation. Stem Cells 28 (2), 357–364. 10.1002/stem.288 20039258 PMC2837600

[B83] Huang R. Qin C. Wang J. Hu Y. Zheng G. Qiu G. (2019a). Differential effects of extracellular vesicles from aging and young mesenchymal stem cells in acute lung injury. Aging-Us 11 (18), 7996–8014. 10.18632/aging.102314 PMC6781978 31575829

[B84] Huang Y. Wu Q. Paul Kwong Hang Tam (2022). Immunomodulatory mechanisms of mesenchymal stem cells and their potential clinical applications. Int. J. Mol. Sci. 23 (17), 10023. 10.3390/ijms231710023 36077421 PMC9456387

[B85] Huang Y. C. Lai L. C. (2019b). The potential roles of stem cell-derived extracellular vesicles as a therapeutic tool. Ann. Transl. Med. 7 (22), 693. 10.21037/atm.2019.11.66 31930094 PMC6944607

[B86] Humrich J. Y. Riemekasten. G. (2016). Restoring regulation - IL-2 therapy in systemic lupus erythematosus. Expert Rev. Clin. Immunol. 12 (11), 1153–1160. 10.1080/1744666x.2016.1199957 27283871

[B87] Jackson M. V. Morrison T. J. Doherty D. F. McAuley D. F. Matthay M. A. Kissenpfennig A. (2016). Mitochondrial transfer via tunneling nanotubes is an important mechanism by which mesenchymal stem cells enhance macrophage phagocytosis in the *in vitro* and *in vivo* models of ARDS. Stem Cells 34 (8), 2210–2223. 10.1002/stem.2372 27059413 PMC4982045

[B88] Ji J. Fu T. Dong C. Zhu W. Yang J. Kong X. (2019). Targeting HMGB1 by ethyl pyruvate ameliorates systemic lupus erythematosus and reverses the senescent phenotype of bone marrow-mesenchymal stem cells. Aging-Us 11 (13), 4338–4353. 10.18632/aging.102052 PMC6660056 31303606

[B89] Ji J. Wu Y. Meng Y. Zhang L. Feng G. Xia Y. (2017). JAK-STAT signaling mediates the senescence of bone marrow-mesenchymal stem cells from systemic lupus erythematosus patients. Acta Biochimica Biophysica Sinica 49 (3), 208–215. 10.1093/abbs/gmw134 28177455

[B90] Jiang X. Li W. Ge L. Lu M. (2023). Mesenchymal stem cell senescence during aging:from mechanisms to rejuvenation strategies. Aging Dis. 14 (5), 1651–1676. 10.14336/ad.2023.0208 37196126 PMC10529739

[B91] Jing H. Su X. Gao Bo Yi S. Chen Ji Deng Z. (2022). Epigenetic inhibition of Wnt pathway suppresses osteogenic differentiation of BMSCs during osteoporosis. Cell. Death Dis. 13 (2). 10.1038/s41419-022-04616-z PMC8885658 35228514

[B92] Johnson S. C. Rabinovitch P. S. Kaeberlein M. (2013). mTOR is a key modulator of ageing and age-related disease. Nature 493 (7432), 338–345. 10.1038/nature11861 23325216 PMC3687363

[B93] Kamal N. S. M. Safuan S. Shamsuddin S. Foroozandeh P. (2020). Aging of the cells: insight into cellular senescence and detection Methods. Eur. J. Cell. Biol. 99 (6), 151108. 10.1016/j.ejcb.2020.151108 32800277

[B94] Kang D. Shin J. Cho Y. Kim H.-S. Gu Y.-R. Kim H. (2019). Stress-activated miR-204 governs senescent phenotypes of chondrocytes to promote osteoarthritis development. Sci. Transl. Med. 11 (486), eaar6659. 10.1126/scitranslmed.aar6659 30944169

[B95] Kapetanou M. Chondrogianni N. Petrakis S. George K. EfstathiosGonos S. (2017b). Proteasome activation enhances stemness and lifespan of human mesenchymal stem cells. Free Radic. Biol. Med. 103, 226–235. 10.1016/j.freeradbiomed.2016.12.035 28034832

[B96] Kapetanou M. Chondrogianni N. Petrakis S. Koliakos G. Gonos E. S. (2017a). Proteasome activation enhances stemness and lifespan of human mesenchymal stem cells. Free Radic. Biol. Med. 103, 226–235. 10.1016/j.freeradbiomed.2016.12.035 28034832

[B97] Karakoc M. Altindag O. Keles H. Soran N. Selek S. (2007). Serum oxidative - antioxidative status in patients with ankylosing spondilitis. Rheumatol. Int. 27 (12), 1131–1134. 10.1007/s00296-007-0352-3 17443328

[B98] Khodayari S. Hamid K. Amiri A. Z. Eslami M. Farhud D. Hescheler J. (2019). Inflammatory microenvironment of acute myocardial infarction prevents regeneration of heart with stem cells therapy. Cell. physiology Biochem. Int. J. Exp. Cell. physiology, Biochem. Pharmacol. 53 (5), 887–909. 10.33594/000000180 31749350

[B99] Khorraminejad-Shirazi M. Sani M. Talaei-Khozani T. Dorvash M. Mirzaei M. Faghihi M. A. (2020). AICAR and nicotinamide treatment synergistically augment the proliferation and attenuate senescence-associated changes in mesenchymal stromal cells. Stem Cell. Res. Ther. 11 (1), 45. 10.1186/s13287-020-1565-6 32014016 PMC6998366

[B100] Kim C. Park J.-M. Song Y. Kim S. Moon J. (2019). HIF1 alpha-mediated AIMP3 suppression delays stem cell aging via the induction of autophagy. Aging Cell. 18 (2), e12909. 10.1111/acel.12909 30706629 PMC6413650

[B101] Kim D. W. Jeong H. S. Kim E. Lee H. Choi C. H. Lee S. J. (2022). Oral delivery of stem-cell-loaded hydrogel microcapsules restores gut inflammation and microbiota. J. Control Release 347, 508–520. 10.1016/j.jconrel.2022.05.028 35597403

[B102] Kim J.-A. Shim J.-S. Lee G.-Y. Yim H. W. Kim T.-M. Kim M. (2015). Microenvironmental remodeling as a parameter and prognostic factor of heterogeneous leukemogenesis in acute myelogenous leukemia. Cancer Res. 75 (11), 2222–2231. 10.1158/0008-5472.Can-14-3379 25791383

[B103] Kim J. H. Oh S.-H. Kim E.-J. Park S. J. Sung P. H. Cheon J. H. (2012). The role of myofibroblasts in upregulation of S100A8 and S100A9 and the differentiation of myeloid cells in the colorectal cancer microenvironment. Biochem. Biophysical Res. Commun. 423 (1), 60–66. 10.1016/j.bbrc.2012.05.081 22634002

[B104] Kim Y. G. Choi J. Kim K. (2020). Mesenchymal stem cell-derived exosomes for effective cartilage tissue repair and treatment of osteoarthritis. Biotechnol. J. 15 (12), e2000082. 10.1002/biot.202000082 32559340

[B105] Kong C.-M. Subramanian A. Biswas A. Walter S. Chong Y.-S. Bongso A. (2019). Changes in stemness properties, differentiation potential, oxidative stress, senescence and mitochondrial function in Wharton's jelly stem cells of umbilical cords of mothers with gestational diabetes mellitus. Stem Cell. Rev. Rep. 15 (3), 415–426. 10.1007/s12015-019-9872-y 30645713

[B106] Korolchuk V. I. Miwa S. Carroll B. von Zglinicki T. (2017). Mitochondria in cell senescence: is mitophagy the weakest link? Ebiomedicine 21, 7–13. 10.1016/j.ebiom.2017.03.020 28330601 PMC5514379

[B107] Kowald A. Passos J. F. Kirkwood T. B. L. (2020). On the evolution of cellular senescence. Aging Cell. 19 (12), e13270. 10.1111/acel.13270 33166065 PMC7744960

[B108] Kumar A. Anand T. Bhattacharyya J. Sharma A. Grace Jaganathan B. (2018). K562 chronic myeloid leukemia cells modify osteogenic differentiation and gene expression of bone marrow stromal cells. J. Cell. Commun. Signal. 12 (2), 441–450. 10.1007/s12079-017-0412-8 28963654 PMC5910323

[B109] Kumar S. K. Rajkumar V. Kyle R. A. van Duin M. Sonneveld P. Mateos M. V. (2017). Multiple myeloma. Nat. Rev. Dis. Prim. 3, 17046. 10.1038/nrdp.2017.46 28726797

[B110] Kutyna M. M. Kok C. H. Lim Y. Ngoc Hoa Tran E. Campbell D. Paton S. (2022). A senescence stress secretome is a hallmark of therapy-related myeloid neoplasm stromal tissue occurring soon after cytotoxic exposure. Leukemia 36 (11), 2678–2689. 10.1038/s41375-022-01686-y 36038666 PMC9613466

[B111] Le Y. Fraineau S. Priya C. Mitchell S. Brand M. Lavoie J. R. (2016). Adipogenic mesenchymal stromal cells from bone marrow and their hematopoietic supportive role: towards understanding the permissive marrow microenvironment in acute myeloid leukemia. Stem Cell. Rev. Rep. 12 (2), 235–244. 10.1007/s12015-015-9639-z 26649729

[B112] Lee G. R. (2018). The balance of Th17 versus Treg cells in autoimmunity. Int. J. Mol. Sci. 19 (3), 730. 10.3390/ijms19030730 29510522 PMC5877591

[B113] Lehmann J. Narcisi R. Franceschini N. Chatzivasileiou D. Boer C. G. Koevoet W. (2022). WNT/beta-catenin signalling interrupts a senescence-induction cascade in human mesenchymal stem cells that restricts their expansion. Cell. Mol. Life Sci. 79 (2), 82. 10.1007/s00018-021-04035-x 35048158 PMC8770385

[B114] Li X. Hong Y. He H. Jiang G. You W. Liang X. (2019). FGF21 mediates mesenchymal stem cell senescence via regulation of mitochondrial dynamics. Oxidative Med. Cell. Longev. 2019, 4915149. 10.1155/2019/4915149 PMC6501200 31178962

[B115] Li X. Wang X. Zhang C. Wang J. Wang S. Hu L. (2022). Dysfunction of metabolic activity of bone marrow mesenchymal stem cells in aged mice. Cell. Prolif. 55 (3), e13191. 10.1111/cpr.13191 35088483 PMC8891618

[B116] Li Y. Lu L. Xie Y. Chen X. Tian L. Liang Y. (2020). Interleukin-6 knockout inhibits senescence of bone mesenchymal stem cells in high-fat diet-induced bone loss. Front. Endocrinol. (Lausanne) 11, 622950. 10.3389/fendo.2020.622950 33679606 PMC7933660

[B117] Li Y. Wu Q. Wang Y. Li L. Bu H. Bao J. (2017). Senescence of mesenchymal stem cells (Review). Int. J. Mol. Med. 39 (4), 775–782. 10.3892/ijmm.2017.2912 28290609

[B118] Lin H. Lunetta K. L. Zhao Q. Rong J. Benjamin E. J. Mendelson M. M. (2017). Transcriptome-wide association study of inflammatory biologic age. Aging (Albany NY) 9 (11), 2288–2301. 10.18632/aging.101321 29135455 PMC5723687

[B119] Lin M. Liu X. Zheng H. Huang X. Wu Yu Huang A. (2020). IGF-1 enhances BMSC viability, migration, and anti-apoptosis in myocardial infarction via secreted frizzled-related protein 2 pathway. Stem Cell. Res. Ther. 11 (1), 22. 10.1186/s13287-019-1544-y 31918758 PMC6953226

[B120] Liu C.-H. Sengupta R. Chen C.-H. Hung K.-H. Chou C.-T. Chen I.-Ho (2019). HLA-B27-mediated activation of TNAP phosphatase promotes pathogenic syndesmophyte formation in ankylosing spondylitis. J. Clin. Investigation 129 (12), 5357–5373. 10.1172/jci125212 PMC6877322 31682238

[B121] Liu J. Ding Y. Liu Z. Liang X. (2020a). Senescence in mesenchymal stem cells: functional alterations, molecular mechanisms, and rejuvenation strategies. Front. Cell. Dev. Biol. 8, 258. 10.3389/fcell.2020.00258 32478063 PMC7232554

[B122] Liu J. Gao J. Liang Z. Gao C. Niu Q. Wu F. (2022a). Mesenchymal stem cells and their microenvironment. Stem Cell. Res. Ther. 13 (1), 429. 10.1186/s13287-022-02985-y 35987711 PMC9391632

[B123] Liu L. DiGirolamo C. M. Navarro P. Blasco M. A. Keefe D. L. (2004). Telomerase deficiency impairs differentiation of mesenchymal stem cells. Exp. Cell. Res. 294 (1), 1–8. 10.1016/j.yexcr.2003.10.031 14980495

[B124] Liu M. Lei H. Dong P. Fu X. Yang Z. Yang Y. (2017). Adipose-derived mesenchymal stem cells from the elderly exhibit decreased migration and differentiation abilities with senescent properties. Cell. Transplant. 26 (9), 1505–1519. 10.1177/0963689717721221 29113467 PMC5680952

[B125] Liu W. Rong Y. Wang J. Zheng Z. Ge X. Ji C. (2020b). Exosome-shuttled miR-216a-5p from hypoxic preconditioned mesenchymal stem cells repair traumatic spinal cord injury by shifting microglial M1/M2 polarization. J. Neuroinflammation 17 (1), 47. 10.1186/s12974-020-1726-7 32019561 PMC7001326

[B126] Liu X. P. Zhan Y. B. Xu W. X. Liu L. X. Liu X. Y. Da J. L. (2022b). Characterization of transcriptional landscape in bone marrow-derived mesenchymal stromal cells treated with aspirin by RNA-seq. Peerj 10, e12819. 10.7717/peerj.12819 35127290 PMC8793730

[B127] Liu Y. Schwam J. Chen Q. (2022c). Senescence-associated cell transition and interaction (sactai): a proposed mechanism for tissue aging, repair, and degeneration. Cells 11 (7), 1089. 10.3390/cells11071089 35406653 PMC8997723

[B128] Lopez-Otin C. Blasco M. A. Partridge L. Serrano M. Guido K. (2013). The hallmarks of aging. Cell. 153 (6), 1194–1217. 10.1016/j.cell.2013.05.039 23746838 PMC3836174

[B129] Lozono-Torres B. Alejandro E.-F. Rovira M. Orzaez M. Serrano M. Martinez-Manez R. (2019). The chemistry of senescence. Nat. Rev. Chem. 3 (7), 426–441. 10.1038/s41570-019-0108-0

[B130] Luo Z. Wu F. Xue E. Huang L. Yan P. Pan X. (2019). Hypoxia preconditioning promotes bone marrow mesenchymal stem cells survival by inducing HIF-1 alpha in injured neuronal cells derived exosomes culture system. Cell. Death Dis. 10, 134. 10.1038/s41419-019-1410-y 30755595 PMC6372680

[B131] Ma C. Pi C. Yang Y. Lin L. Shi Y. Li Y. (2017). Nampt expression decreases age-related senescence in rat bone marrow mesenchymal stem cells by targeting Sirt1. PLoS One 12 (1), e0170930. 10.1371/journal.pone.0170930 28125705 PMC5268649

[B132] Ma C. Sun Y. Pi C. Wang H. Sun H. Xiao Yu (2020). Sirt3 attenuates oxidative stress damage and rescues cellular senescence in rat bone marrow mesenchymal stem cells by targeting superoxide dismutase 2. Front. Cell. Dev. Biol. 8, 599376. 10.3389/fcell.2020.599376 33330487 PMC7718008

[B133] Mareschi K. Ferrero I. Rustichelli D. Aschero S. Gammaitoni L. Aglietta M. (2006). Expansion of mesenchymal stem cells isolated from pediatric and adult donor bone marrow. J. Cell. Biochem. 97 (4), 744–754. 10.1002/jcb.20681 16229018

[B134] Marinkovic M. Dai Q. Gonzalez A. O. Tran O. N. Block T. J. Harris S. E. (2022). Matrix-bound Cyr61/CCN1 is required to retain the properties of the bone marrow mesenchymal stem cell niche but is depleted with aging. Matrix Biol. 111, 108–132. 10.1016/j.matbio.2022.06.004 35752272 PMC10069241

[B135] Marote A. Santos D. Mendes-Pinheiro B. Serre-Miranda C. Anjo S. I. Vieira J. (2023). Cellular aging secretes: a comparison of bone-marrow-derived and induced mesenchymal stem cells and their secretome over long-term culture. Stem Cell. Rev. Rep. 19 (1), 248–263. 10.1007/s12015-022-10453-6 36152233

[B136] Massa M. Croce S. Campanelli R. Abbà C. Lenta E. Valsecchi C. (2020). Clinical applications of mesenchymal stem/stromal cell derived extracellular vesicles: therapeutic potential of an acellular product. Diagn. (Basel) 10 (12), 999. 10.3390/diagnostics10120999 PMC7760121 33255416

[B137] Mato-Basalo R. Morente-Lopez M. Arntz O. J. van de Loo F. A. J. Fafian-Labora J. Arufe M. C. (2021). Therapeutic potential for regulation of the nuclear factor kappa-B transcription factor p65 to prevent cellular senescence and activation of pro-inflammatory in mesenchymal stem cells. Int. J. Mol. Sci. 22 (7), 3367. 10.3390/ijms22073367 33805981 PMC8038109

[B138] Mattiucci D. Maurizi G. Leoni P. Poloni A. (2018). Aging- and senescence-associated changes of mesenchymal stromal cells in myelodysplastic syndromes. Cell. Transplant. 27 (5), 754–764. 10.1177/0963689717745890 29682980 PMC6047275

[B139] Meyerrose T. Olson S. Pontow S. Kalomoiris S. Jung Y. Annett G. (2010). Mesenchymal stem cells for the sustained *in vivo* delivery of bioactive factors. Adv. Drug Deliv. Rev. 62 (12), 1167–1174. 10.1016/j.addr.2010.09.013 20920540 PMC3815452

[B140] Miao C. Lei M. Hu W. Han S. Wang Qi (2017). A brief review: the therapeutic potential of bone marrow mesenchymal stem cells in myocardial infarction. Stem Cell. Res. Ther. 8, 242. 10.1186/s13287-017-0697-9 29096705 PMC5667518

[B141] Miura Y. Endo K. Komori K. Sekiya I. (2022). Clearance of senescent cells with ABT-263 improves biological functions of synovial mesenchymal stem cells from osteoarthritis patients. Stem Cell. Res. Ther. 13 (1), 222. 10.1186/s13287-022-02901-4 35658936 PMC9166575

[B142] Moyzis R. K. Buckingham J. M. Cram L. S. Dani M. Deaven L. L. Jones M. D. (1988). A highly conserved repetitive DNA sequence, (TTAGGG)n, present at the telomeres of human chromosomes. Proc. Natl. Acad. Sci. U. S. A. 85 (18), 6622–6626. 10.1073/pnas.85.18.6622 3413114 PMC282029

[B143] Musavi M. Kohram F. Abasi M. Bolandi Z. Ajoudanian M. Mohammadi-Yeganeh S. (2019). Rn7SK small nuclear RNA is involved in cellular senescence. J. Cell. Physiology 234 (8), 14234–14245. 10.1002/jcp.28119 PMC13483005 30637716

[B144] Najafi H. Abolmaali S. S. Heidari R. Valizadeh H. Tamaddon A. M. Azarpira N. (2022). Integrin receptor-binding nanofibrous peptide hydrogel for combined mesenchymal stem cell therapy and nitric oxide delivery in renal ischemia/reperfusion injury. Stem Cell. Res. Ther. 13 (1), 344. 10.1186/s13287-022-03045-1 35883125 PMC9327234

[B145] Nowak B. Rogujski P. Janowski M. Lukomska B. Andrzejewska A. (2021). Mesenchymal stem cells in glioblastoma therapy and progression: how one cell does it all. Biochim. Biophys. Acta Rev. Cancer 1876 (1), 188582. 10.1016/j.bbcan.2021.188582 34144129

[B146] Oja S. Komulainen P. Penttilä A. Nystedt J. Korhonen M. (2018). Automated image analysis detects aging in clinical-grade mesenchymal stromal cell cultures. Stem Cell. Res. Ther. 9 (1), 6. 10.1186/s13287-017-0740-x 29321040 PMC5763576

[B147] Oka T. Hikoso S. Yamaguchi O. Taneike M. Takeda T. Tamai T. (2012). Mitochondrial DNA that escapes from autophagy causes inflammation and heart failure. Nature 485 (7397), 251–255. 10.1038/nature10992 22535248 PMC3378041

[B148] Onyiah J. C. Colgan S. P. (2016). Cytokine responses and epithelial function in the intestinal mucosa. Cell. Mol. Life Sci. 73 (22), 4203–4212. 10.1007/s00018-016-2289-8 27271753 PMC5056122

[B149] Ou H.-L. Schumacher B. (2018). DNA damage responses and p53 in the aging process. Blood 131 (5), 488–495. 10.1182/blood-2017-07-746396 29141944 PMC6839964

[B150] Panés J. García-Olmo D. Van Assche G. Colombel J. F. Reinisch W. Baumgart D. C. (2018). Long-term efficacy and safety of stem cell therapy (Cx601) for complex perianal fistulas in patients with Crohn's disease. Gastroenterology 154 (5), 1334–1342.e4. 10.1053/j.gastro.2017.12.020 29277560

[B151] Papait A. Stefani F. R. Cargnoni A. Magatti M. Parolini O. Silini A. R. (2020). The multifaceted roles of MSCs in the tumor microenvironment: interactions with immune cells and exploitation for therapy. Front. Cell. Dev. Biol. 8, 447. 10.3389/fcell.2020.00447 32637408 PMC7317293

[B152] Parsch D. Fellenberg J. Brümmendorf T. H. Eschlbeck A. M. Richter W. (2004). Telomere length and telomerase activity during expansion and differentiation of human mesenchymal stem cells and chondrocytes. J. Mol. Med. Berl. 82 (1), 49–55. 10.1007/s00109-003-0506-z 14647922

[B153] Peck S. H. Bendigo J. R. Tobias J. W. Dodge G. R. Malhotra N. R. Mauck R. L. (2021). Hypoxic preconditioning enhances bone marrow-derived mesenchymal stem cell survival in a low oxygen and nutrient-limited 3D microenvironment. Cartilage 12 (4), 512–525. 10.1177/1947603519841675 30971109 PMC8461160

[B154] Peffers M. J. Collins J. Fang Y. Goljanek-Whysall K. Rushton M. Loughlin J. (2016). Age-related changes in mesenchymal stem cells identified using a multi-omics approach. Eur. Cell. Mater 31, 136–159. 10.22203/ecm.v031a10 26853623

[B155] Pei F. Ma L. Jing J. Feng J. Yuan Y. Guo T. (2023). Sensory nerve niche regulates mesenchymal stem cell homeostasis via FGF/mTOR/autophagy axis. Nat. Commun. 14 (1), 344. 10.1038/s41467-023-35977-4 36670126 PMC9859800

[B156] Peng X. Zhou X. Yin Y. Luo B. Liu Y. Cheng Y. (2022). Inflammatory microenvironment accelerates bone marrow mesenchymal stem cell aging. Front. Bioeng. Biotechnol. 10, 870324. 10.3389/fbioe.2022.870324 35646835 PMC9133389

[B157] Phelps J. Hart D. A. Mitha A. P. Duncan N. A. Sen A. (2023). Physiological oxygen conditions enhance the angiogenic properties of extracellular vesicles from human mesenchymal stem cells. Stem Cell. Res. Ther. 14 (1), 218. 10.1186/s13287-023-03439-9 37612731 PMC10463845

[B158] Pi C. Cao Ma Wang H. Sun H. Xiao Yu Gao X. (2021). MiR-34a suppression targets Nampt to ameliorate bone marrow mesenchymal stem cell senescence by regulating NAD(+)-Sirt1 pathway. Stem Cell. Res. Ther. 12 (1), 271. 10.1186/s13287-021-02339-0 33957971 PMC8101138

[B159] Pignolo R. J. Passos J. F. Khosla S. Tchkonia T. Kirkland J. L. (2020). Reducing senescent cell burden in aging and disease. Trends Mol. Med. 26 (7), 630–638. 10.1016/j.molmed.2020.03.005 32589933 PMC7857028

[B160] Pignolo R. J. Suda R. K. McMillan E. A. Shen J. Lee S.-H. Choi Y. (2008). Defects in telomere maintenance molecules impair osteoblast differentiation and promote osteoporosis. Aging Cell. 7 (1), 23–31. 10.1111/j.1474-9726.2007.00350.x 18028256 PMC2394673

[B161] Pittenger M. F. Discher D. E. Péault B. M. Phinney D. G. Hare J. M. Caplan A. I. (2019). Mesenchymal stem cell perspective: cell biology to clinical progress. NPJ Regen. Med. 4, 22. 10.1038/s41536-019-0083-6 31815001 PMC6889290

[B162] Prattichizzo F. De Nigris V. Mancuso E. Spiga R. Giuliani A. Matacchione G. (2018). Short-term sustained hyperglycaemia fosters an archetypal senescence-associated secretory phenotype in endothelial cells and macrophages. Redox Biol. 15, 170–181. 10.1016/j.redox.2017.12.001 29253812 PMC5735298

[B163] Psaroudis R. T. Singh U. Lora M. Jeon P. Boursiquot A. Stochaj U. (2022). CD26 is a senescence marker associated with reduced immunopotency of human adipose tissue-derived multipotent mesenchymal stromal cells. Stem Cell. Res. Ther. 13 (1), 358. 10.1186/s13287-022-03026-4 35883188 PMC9327293

[B164] Qi Y. Zhu T. Zhang T. Wang Xi Li W. Chen D. (2021). M1 macrophage-derived exosomes transfer miR-222 to induce bone marrow mesenchymal stem cell apoptosis. Lab. Investig. 101 (10), 1318–1326. 10.1038/s41374-021-00622-5 34230646

[B165] Qiao Q. Liu X. Yang T. Cui K. Kong L. Yang C. (2021). Nanomedicine for acute respiratory distress syndrome: the latest application, targeting strategy, and rational design. Acta Pharm. Sin. B 11 (10), 3060–3091. 10.1016/j.apsb.2021.04.023 33977080 PMC8102084

[B166] Ray P. D. Huang B. W. Tsuji Y. (2012). Reactive oxygen species (ROS) homeostasis and redox regulation in cellular signaling. Cell. Signal 24 (5), 981–990. 10.1016/j.cellsig.2012.01.008 22286106 PMC3454471

[B167] Redondo J. Sarkar P. Kemp K. Heesom K. J. Wilkins A. Scolding N. J. (2018b). Dysregulation of mesenchymal stromal cell antioxidant responses in progressive multiple sclerosis. Stem Cells Transl. Med. 7 (10), 748–758. 10.1002/sctm.18-0045 30063300 PMC6186266

[B168] Redondo J. Sarkar P. Kemp K. Virgo P. F. Pawade J. Norton A. (2018a). Reduced cellularity of bone marrow in multiple sclerosis with decreased MSC expansion potential and premature ageing *in vitro* . Mult. Scler. 24 (7), 919–931. 10.1177/1352458517711276 28548004 PMC6029147

[B169] Redondo J. Sarkar P. Kemp K. Virgo P. F. Pawade J. Norton A. (2018c). Reduced cellularity of bone marrow in multiple sclerosis with decreased MSC expansion potential and premature ageing *in vitro* . Multiple Scler. J. 24 (7), 919–931. 10.1177/1352458517711276 PMC6029147 28548004

[B170] Rehman S.Ur Shah S. A. Ali T. Chung J.Il Kim M.Ok (2017). Anthocyanins reversed D-galactose-induced oxidative stress and neuroinflammation mediated cognitive impairment in adult rats. Mol. Neurobiol. 54 (1), 255–271. 10.1007/s12035-015-9604-5 26738855

[B171] Ricci S. Cacialli P. (2021). Stem cell research tools in human metabolic disorders: an overview. Cells 10 (10), 2681. 10.3390/cells10102681 34685661 PMC8534517

[B172] Ridzuan N. Al Abbar A. Yip W. K. Maqbool M. Ramasamy R. (2016). Characterization and expression of senescence marker in prolonged passages of rat bone marrow-derived mesenchymal stem cells. Stem Cells Int. 2016, 8487264. 10.1155/2016/8487264 27579045 PMC4989133

[B173] Ritschka B. Storer M. Mas A. Heinzmann F. Ortells M. C. Morton J. P. (2017). The senescence-associated secretory phenotype induces cellular plasticity and tissue regeneration. Genes. & Dev. 31 (2), 172–183. 10.1101/gad.290635.116 28143833 PMC5322731

[B174] Rizzo M. G. Best T. M. Huard J. Philippon M. Hornicek F. Duan Z. (2023). Therapeutic perspectives for inflammation and senescence in osteoarthritis using mesenchymal stem cells, mesenchymal stem cell-derived extracellular vesicles and senolytic agents. Cells 12 (10), 1421. 10.3390/cells12101421 37408255 PMC10217382

[B175] Salminen A. Kaarniranta K. Kauppinen A. (2016). Age-related changes in AMPK activation: role for AMPK phosphatases and inhibitory phosphorylation by upstream signaling pathways. Ageing Res. Rev. 28, 15–26. 10.1016/j.arr.2016.04.003 27060201

[B176] Seok J. Jung H. S. Park S. Lee J.Ok Kim C. J. Kim Gi J. (2020). Alteration of fatty acid oxidation by increased CPT1A on replicative senescence of placenta-derived mesenchymal stem cells. Stem Cell. Res. Ther. 11 (1), 1. 10.1186/s13287-019-1471-y 31900237 PMC6941254

[B177] Shen J. Zhu X. Liu H. (2020). MiR-483 induces senescence of human adipose-derived mesenchymal stem cells through IGF1 inhibition. Aging-Us 12 (15), 15756–15770. 10.18632/aging.103818 PMC7467354 32805717

[B178] Shi L. Zhao Y. Fei C. Guo J. Jia Y. Wu D. (2019). Cellular senescence induced by S100A9 in mesenchymal stromal cells through NLRP3 inflammasome activation. Aging-Us 11 (21), 9626–9642. 10.18632/aging.102409 PMC6874461 31727865

[B179] Sim S. W. Weinstein D. A. Lee Y. M. Jun H. S. (2020). Glycogen storage disease type Ib: role of glucose-6-phosphate transporter in cell metabolism and function. FEBS Lett. 594 (1), 3–18. 10.1002/1873-3468.13666 31705665

[B180] Siraj Y. Galderisi U. Alessio N. (2023). Senescence induces fundamental changes in the secretome of mesenchymal stromal cells (MSCs): implications for the therapeutic use of MSCs and their derivates. Front. Bioeng. Biotechnol. 11, 1148761. 10.3389/fbioe.2023.1148761 37229499 PMC10203235

[B181] Soares R. O. S. Losada D. M. Jordani M. C. Evora P. Castro-e-Silva O. (2019). Ischemia/reperfusion injury revisited: an overview of the latest pharmacological strategies. Int. J. Mol. Sci. 20 (20), 5034. 10.3390/ijms20205034 31614478 PMC6834141

[B182] Sokal E. M. Lombard C. Mazza G. (2015). Mesenchymal stem cell treatment for hemophilia: a review of current knowledge. J. Thromb. Haemost. 13 (Suppl. 1), S161–S166. 10.1111/jth.12933 26149017

[B183] Specchio N. Ferretti A. Trivisano M. Pietrafusa N. Pepi C. Calabrese C. (2021). Neuronal ceroid lipofuscinosis: potential for targeted therapy. Drugs 81 (1), 101–123. 10.1007/s40265-020-01440-7 33242182

[B184] Sui B. Hu C. Jin Y. (2016). Mitochondrial metabolic failure in telomere attrition-provoked aging of bone marrow mesenchymal stem cells. Biogerontology 17 (2), 267–279. 10.1007/s10522-015-9609-5 26392399

[B185] Sun F. Sun Y. Wu F. Xu W. Qian H. (2022a). Mesenchymal stem cell-derived extracellular vesicles: a potential therapy for diabetes mellitus and diabetic complications. Pharmaceutics 14 (10), 2208. 10.3390/pharmaceutics14102208 36297643 PMC9607185

[B186] Sun S. Xie F. Xu X. Cai Q. Zhang Q. Cui Z. (2018). Advanced oxidation protein products induce S-phase arrest of hepatocytes via the ROS-dependent, beta-catenin-CDK2-mediated pathway. Redox Biol. 14, 338–353. 10.1016/j.redox.2017.09.011 29032312 PMC5975226

[B187] Sun Y. Zhang H. Qiu T. Liao L. Su X. (2023). Epigenetic regulation of mesenchymal stem cell aging through histone modifications. Genes. Dis. 10 (6), 2443–2456. 10.1016/j.gendis.2022.10.030 37554203 PMC10404871

[B188] Sun Z. Hou X. Zhang J. Li J. Wu P. Yan L. (2022b). Diagnostic and therapeutic roles of extracellular vesicles in aging-related diseases. Oxidative Med. Cell. Longev. 2022, 6742792. 10.1155/2022/6742792 PMC9377967 35979398

[B189] Tan L. Liu X. Dou H. Hou Y. (2022). Characteristics and regulation of mesenchymal stem cell plasticity by the microenvironment - specific factors involved in the regulation of MSC plasticity. Genes. Dis. 9 (2), 296–309. 10.1016/j.gendis.2020.10.006 35224147 PMC8843883

[B190] Tencerova M. Frost M. Figeac F. Nielsen T. K. Ali D. Lauterlein J.-J. L. 2019. "Obesity-associated hypermetabolism and accelerated senescence of bone marrow stromal stem cells suggest a potential mechanism for bone fragility." Cell. Rep. 27 (7):2050–2062. 10.1016/j.celrep.2019.04.066 31091445

[B191] Tian D. Liu J. Chen L. Zhu B. Jing J. (2020). The protective effects of PI3K/Akt pathway on human nucleus pulposus mesenchymal stem cells against hypoxia and nutrition deficiency. J. Orthop. Surg. Res. 15 (1), 29. 10.1186/s13018-020-1551-9 31992313 PMC6988348

[B192] Trani J. P. Chevalier R. Caron L. El Yazidi C. Broucqsault N. Toury L. (2022). Mesenchymal stem cells derived from patients with premature aging syndromes display hallmarks of physiological aging. Life Sci. Alliance 5 (12), e202201501. 10.26508/lsa.202201501 36104080 PMC9475049

[B193] Truong N. C. Bui K. H. T. Pham P. V. 2019. "Characterization of senescence of human adipose-derived stem cells after long-term expansion." In Tissue engineering and regenerative medicine, edited by P. VanPham, 109–128. 10.1007/5584_2018_235 30242785

[B194] Vanegas N.-D. P. Ruiz-Aparicio P. F. Uribe G. I. Adriana L.-B. Vernot J.-P. (2021). Leukemia-induced cellular senescence and stemness alterations in mesenchymal stem cells are reversible upon withdrawal of B-cell acute lymphoblastic leukemia cells. Int. J. Mol. Sci. 22 (15), 8166. 10.3390/ijms22158166 34360930 PMC8348535

[B195] Varderidou-Minasian S. Lorenowicz M. J. (2020). Mesenchymal stromal/stem cell-derived extracellular vesicles in tissue repair: challenges and opportunities. Theranostics 10 (13), 5979–5997. 10.7150/thno.40122 32483432 PMC7254996

[B196] Venkatachalam K. Venkatesan B. Valente A. J. Melby P. C. Nandish S. Reusch J. E. (2009). WISP1, a pro-mitogenic, pro-survival factor, mediates tumor necrosis factor-alpha (TNF-alpha)-stimulated cardiac fibroblast proliferation but inhibits TNF-alpha-induced cardiomyocyte death. J. Biol. Chem. 284 (21), 14414–14427. 10.1074/jbc.M809757200 19339243 PMC2682890

[B197] Vernot J.-P. Bonilla X. Rodriguez-Pardo V. Vanegas N.-D. P. (2017). Phenotypic and functional alterations of hematopoietic stem and progenitor cells in an *in vitro* leukemia-induced microenvironment. Int. J. Mol. Sci. 18 (2), 199. 10.3390/ijms18020199 28216566 PMC5343770

[B198] Vida C. Martinez de Toda I. Cruces J. Garrido A. Gonzalez-Sanchez M. De la Fuente M. (2017). Role of macrophages in age-related oxidative stress and lipofuscin accumulation in mice. Redox Biol. 12, 423–437. 10.1016/j.redox.2017.03.005 28319893 PMC5357673

[B199] Wang F. Guo J. Wang S. Wang Y. Chen J. Hu Y. (2022b). B-cell lymphoma-3 controls mesenchymal stem cell commitment and senescence during skeletal aging. Clin. Transl. Med. 12 (7), e955. 10.1002/ctm2.955 35804493 PMC9270574

[B200] Wang F. X. Guo J. W. Wang S. C. Wang Y. L. Chen J. Hu Y. (2022a). B-cell lymphoma-3 controls mesenchymal stem cell commitment and senescence during skeletal aging. Clin. Transl. Med. 12 (7), e955. 10.1002/ctm2.955 35804493 PMC9270574

[B201] Wang H. Sun Y. Pi C. Xiao Yu Gao X. Zhang C. (2022c). Nicotinamide mononucleotide supplementation improves mitochondrial dysfunction and rescues cellular senescence by NAD+/Sirt3 pathway in mesenchymal stem cells. Int. J. Mol. Sci. 23 (23), 14739. 10.3390/ijms232314739 36499074 PMC9738479

[B202] Wang S. Wang Z. Su H. Chen F. Ma M. Yu W. (2021a). Effects of long-term culture on the biological characteristics and RNA profiles of human bone-marrow-derived mesenchymal stem cells. Mol. Ther. Nucleic Acids 26, 557–574. 10.1016/j.omtn.2021.08.013 34631285 PMC8479280

[B203] Wang X. Bootsma H. Kroese F. Dijkstra G. Pringle S. (2020). Senescent stem and transient amplifying cells in Crohn's disease intestine. Inflamm. Bowel Dis. 26 (2), E8–E9. 10.1093/ibd/izz295 31769481 PMC6943680

[B204] Wang X. Ma L. Pei X. Wang H. Tang X. Pei J. F. (2022d). Comprehensive assessment of cellular senescence in the tumor microenvironment. Brief. Bioinform 23 (3), bbac118. 10.1093/bib/bbac118 35419596 PMC9116224

[B205] Wang Y. Sui Y. Lian A. Han X. Liu F. Zuo K. (2021b). PBX1 attenuates hair follicle-derived mesenchymal stem cell senescence and apoptosis by alleviating reactive oxygen species-mediated DNA damage instead of enhancing DNA damage repair. Front. Cell. Dev. Biol. 9, 739868. 10.3389/fcell.2021.739868 34869323 PMC8634257

[B206] Ward M. M. Deodhar A. Gensler L. S. Dubreuil M. Yu D. Khan M. A. (2019). 2019 update of the American College of rheumatology/spondylitis association of America/spondyloarthritis research and treatment network recommendations for the treatment of ankylosing spondylitis and nonradiographic axial spondyloarthritis. Arthritis & Rheumatology 71 (10), 1599–1613. 10.1002/art.41042 31436036 PMC6764882

[B207] Watson J. D. (1972). Origin of concatemeric T7 DNA. Nat. New Biol. 239 (94), 197–201. 10.1038/newbio239197a0 4507727

[B208] Weilner S. Schraml E. Wieser M. Messner P. Schneider K. Wassermann K. (2016). Secreted microvesicular miR-31 inhibits osteogenic differentiation of mesenchymal stem cells. Aging Cell. 15 (4), 744–754. 10.1111/acel.12484 27146333 PMC4933673

[B209] Weng Z. Wang Y. Ouchi T. Liu H. Qiao X. Wu C. (2022). Mesenchymal stem/stromal cell senescence: hallmarks, mechanisms, and combating strategies. Stem Cells Transl. Med. 11 (4), 356–371. 10.1093/stcltm/szac004 35485439 PMC9052415

[B210] Weng Z. Zhang B. Wu C. Yu F. Han B. Li B. (2021). Therapeutic roles of mesenchymal stem cell-derived extracellular vesicles in cancer. J. Hematol. Oncol. 14 (1), 136. 10.1186/s13045-021-01141-y 34479611 PMC8414028

[B211] Wieliczko M. Matuszkiewicz-Rowinska J. (2017). Systemic lupus erythematosus - news 2017. Wiadomosci Lek. 70 (2), 1201–1204. 29533914

[B212] Wong P. F. Dharmani M. Ramasamy T. S. (2023). Senotherapeutics for mesenchymal stem cell senescence and rejuvenation. Drug Discov. Today 28 (1), 103424. 10.1016/j.drudis.2022.103424 36332835

[B213] Wu J. H. Lin T. Gao Y. Li X. M. Yang C. Zhang K. (2022). Long noncoding RNA ZFAS1 suppresses osteogenic differentiation of bone marrow-derived mesenchymal stem cells by upregulating miR-499-EPHA5 axis. Mol. Cell. Endocrinol. 539, 111490. 10.1016/j.mce.2021.111490 34655661

[B214] Wu K. K. (2021). Control of mesenchymal stromal cell senescence by tryptophan metabolites. Int. J. Mol. Sci. 22 (2), 697. 10.3390/ijms22020697 33445766 PMC7828284

[B215] Xia C. Jiang T. Y. Wang Y. H. Chen X. T. Hu Y. Gao Y. H. (2021). The p53/miR-145a Axis promotes cellular senescence and inhibits osteogenic differentiation by targeting cbfb in mesenchymal stem cells. Front. Endocrinol. 11, 609186. 10.3389/fendo.2020.609186 PMC7829338 33505358

[B216] Xiang Q.-yan Tian F. Du X. Xu J. Zhu L.-yuan Guo L.-ling (2020). Postprandial triglyceride-rich lipoproteins-induced premature senescence of adipose-derived mesenchymal stem cells via the SIRT1/p53/Ac-p53/p21 axis through oxidative mechanism. Aging-Us 12 (24), 26080–26094. 10.18632/aging.202298 PMC7803527 33316776

[B217] Xiao F. Peng J. Yang Li Zhou X. Ding Ma Dai L. (2022). Small noncoding RNAome changes during human bone marrow mesenchymal stem cells senescence *in vitro* . Front. Endocrinol. 13, 808223. 10.3389/fendo.2022.808223 PMC9135970 35634512

[B218] Xing J. Ying Y. Mao C. Liu Y. Wang T. Zhao Q. (2018). Hypoxia induces senescence of bone marrow mesenchymal stem cells via altered gut microbiota. Nat. Commun. 9, 2020. 10.1038/s41467-018-04453-9 29789585 PMC5964076

[B219] Xu L. Wang Y. Wang J. Zhai J. Ren L. Zhu G. (2021). Radiation-induced osteocyte senescence alters bone marrow mesenchymal stem cell differentiation potential via paracrine signaling. Int. J. Mol. Sci. 22 (17), 9323. 10.3390/ijms22179323 34502232 PMC8430495

[B220] Xu M. Pirtskhalava T. Farr J. N. Weigand B. M. Palmer A. K. Weivoda M. M. 2018. "Senolytics improve physical function and increase lifespan in old age." Nat. Med. 24 (8):1246–1256. 10.1038/s41591-018-0092-9 29988130 PMC6082705

[B221] Yamaguchi S. Horie N. Satoh K. Ishikawa T. Mori T. Maeda H. (2018). Age of donor of human mesenchymal stem cells affects structural and functional recovery after cell therapy following ischaemic stroke. J. Cereb. Blood Flow Metabolism 38 (7), 1199–1212. 10.1177/0271678x17731964 PMC6434451 28914133

[B222] Yang C. Luo M. Chen Y. You M. Chen Q. (2021). MicroRNAs as important regulators mediate the multiple differentiation of mesenchymal stromal cells. Front. Cell. Dev. Biol. 9, 619842. 10.3389/fcell.2021.619842 34164391 PMC8215576

[B223] Yang C. Yang Y. Ma Li Zhang G.-X. Shi F.-D. Yan Y. (2019). Study of the cytological features of bone marrow mesenchymal stem cells from patients with neuromyelitis optica. Int. J. Mol. Med. 43 (3), 1395–1405. 10.3892/ijmm.2019.4056 30628649 PMC6365084

[B224] Yang G. Fan X. Liu Y. Jie P. Mazhar M. Liu Y. (2023). Immunomodulatory mechanisms and therapeutic potential of mesenchymal stem cells. Stem Cell. Rev. Rep. 19 (5), 1214–1231. 10.1007/s12015-023-10539-9 37058201 PMC10103048

[B225] Yang M. Chen J. Chen Li (2022). The roles of mesenchymal stem cell-derived exosomes in diabetes mellitus and its related complications. Front. Endocrinol. 13, 1027686. 10.3389/fendo.2022.1027686 PMC9633677 36339446

[B226] Yang M. Tong W. Chen H. Deng J. Yang C. Zhang Z. (2018a). Knockdown of insulin-like growth factor 1 exerts a protective effect on hypoxic injury of aged BM-MSCs: role of autophagy. Stem Cell. Res. Ther. 9, 284. 10.1186/s13287-018-1028-5 30359321 PMC6202872

[B227] Yang Y. K. Ogando C. R. Wang See C. Chang T. Y. Barabino G. A. (2018b). Changes in phenotype and differentiation potential of human mesenchymal stem cells aging *in vitro* . Stem Cell. Res. Ther. 9 (1), 131. 10.1186/s13287-018-0876-3 29751774 PMC5948736

[B228] Ye C. Zhang W. Hang K. Chen Mo Hou W. Chen J. (2019). Extracellular IL-37 promotes osteogenic differentiation of human bone marrow mesenchymal stem cells via activation of the PI3K/AKT signaling pathway. Cell. Death Dis. 10, 753. 10.1038/s41419-019-1904-7 31582734 PMC6776644

[B229] Ye G. Xie Z. Zeng H. Wang P. Li J. Zheng G. (2020). Oxidative stress-mediated mitochondrial dysfunction facilitates mesenchymal stem cell senescence in ankylosing spondylitis. Cell. Death Dis. 11 (9), 775. 10.1038/s41419-020-02993-x 32943613 PMC7498590

[B230] Ye M. Huang X. Wu Q. Liu F. (2023). Senescent stromal cells in the tumor microenvironment: victims or accomplices? Cancers (Basel) 15 (7), 1927. 10.3390/cancers15071927 37046588 PMC10093305

[B231] Yi L. Ju Y. He Y. Yin X. Xu Ye Weng T. (2021). Intraperitoneal injection of Desferal® alleviated the age-related bone loss and senescence of bone marrow stromal cells in rats. Stem Cell. Res. Ther. 12 (1), 45. 10.1186/s13287-020-02112-9 33413663 PMC7791659

[B232] Yi S. Lin K. Jiang T. Shao W. Huang C. Jiang B. (2020). NMR-based metabonomic analysis of HUVEC cells during replicative senescence. Aging-Us 12 (4), 3626–3646. 10.18632/aging.102834 PMC7066908 32074082

[B233] Yin M. Zhang Y. Yu H. Xia Li (2021a). Role of hyperglycemia in the senescence of mesenchymal stem cells. Front. Cell. Dev. Biol. 9, 665412. 10.3389/fcell.2021.665412 33968939 PMC8099107

[B234] Yin Y. Chen H. Wang Y. Zhang L. Wang X. (2021b). Roles of extracellular vesicles in the aging microenvironment and age-related diseases. J. Extracell. Vesicles 10 (12), e12154. 10.1002/jev2.12154 34609061 PMC8491204

[B235] Yin Y. Chen H. Wang Y. Zhang L. Wang X. (2021c). Roles of extracellular vesicles in the aging microenvironment and age-related diseases. J. Extracell. Vesicles 10 (12), e12154. 10.1002/jev2.12154 34609061 PMC8491204

[B236] Yu X. Sun H. Gao X. Zhang C. Sun Y. Wang H. (2022a). A comprehensive analysis of age-related metabolomics and transcriptomics reveals metabolic alterations in rat bone marrow mesenchymal stem cells. Aging (Albany NY) 14 (2), 1014–1032. 10.18632/aging.203857 35122680 PMC8833123

[B237] Yu X. Sun H. Gao X. Zhang C. Sun Y. Wang H. (2022b). A comprehensive analysis of age-related metabolomics and transcriptomics reveals metabolic alterations in rat bone marrow mesenchymal stem cells. Aging-Us 14 (2), 1014–1032. 10.18632/aging.203857 PMC8833123 35122680

[B238] Zeng L. Lindstrom M. J. Smith J. A. (2011). Ankylosing spondylitis macrophage production of higher levels of interleukin-23 in response to lipopolysaccharide without induction of a significant unfolded protein response. Arthritis Rheum. 63 (12), 3807–3817. 10.1002/art.30593 22127699 PMC3228355

[B239] Zhai W. Tan J. Russell T. Chen S. McGonagle D. Win Naing M. (2021). Multi-pronged approach to human mesenchymal stromal cells senescence quantification with a focus on label-free methods. Sci. Rep. 11 (1), 1054. 10.1038/s41598-020-79831-9 33441693 PMC7807049

[B240] Zhang B. Zhang J. Zhu D. Kong Ye (2019). Mesenchymal stem cells rejuvenate cardiac muscle after ischemic injury. Aging-Us 11 (1), 63–72. 10.18632/aging.101718 PMC6339792 30613028

[B241] Zhang J. Feng Z. Wei J. Yu Y. Luo J. Zhou J. (2018). Repair of critical-sized mandible defects in aged rat using hypoxia preconditioned BMSCs with up-regulation of hif-1α. Int. J. Biol. Sci. 14 (4), 449–460. 10.7150/ijbs.24158 29725266 PMC5930477

[B242] Zhang L. Chi Y. Wei Y. Zhang W. Wang F. Zhang L. (2021a). Bone marrow-derived mesenchymal stem/stromal cells in patients with acute myeloid leukemia reveal transcriptome alterations and deficiency in cellular vitality. Stem Cell. Res. Ther. 12 (1), 365. 10.1186/s13287-021-02444-0 34174939 PMC8233618

[B243] Zhang L. Zhao Q. Hui C. Wang Z. Hu X. Pan R. (2022a). Acute myeloid leukemia cells educate mesenchymal stromal cells toward an adipogenic differentiation propensity with leukemia promotion capabilities. Adv. Sci. 9 (16), 2105811. 10.1002/advs.202105811 PMC9165478 35686138

[B244] Zhang M. Johnson-Stephenson T. K. Wang W. Wang Y. Jing Li Li L. (2022b). Mesenchymal stem cell-derived exosome-educated macrophages alleviate systemic lupus erythematosus by promoting efferocytosis and recruitment of IL-17(+) regulatory T cell. Stem Cell. Res. Ther. 13 (1), 484. 10.1186/s13287-022-03174-7 36153633 PMC9509559

[B245] Zhang P. Zhang H. Lin J. Xiao T. Xu R. Fu Yu (2020). Insulin impedes osteogenesis of BMSCs by inhibiting autophagy and promoting premature senescence via the TGF-beta 1 pathway. Aging-Us 12 (3), 2084–2100. 10.18632/aging.102723 PMC7041775 32017705

[B246] Zhang Y. Ravikumar M. Ling L. Nurcombe V. Cool S. M. (2021b). Age-related changes in the inflammatory status of human mesenchymal stem cells: implications for cell therapy. Stem Cell. Rep. 16 (4), 694–707. 10.1016/j.stemcr.2021.01.021 PMC8072029 33636113

[B247] Zheng X. Wang Q. Xie Z. Li J. (2021). The elevated level of IL-1α in the bone marrow of aged mice leads to MSC senescence partly by down-regulating Bmi-1. Exp. Gerontol. 148, 111313. 10.1016/j.exger.2021.111313 33740618

[B248] Zheng Y. Wu S. Ke H. Peng S. Hu C. (2023). Secretion of IL-6 and IL-8 in the senescence of bone marrow mesenchymal stem cells is regulated by autophagy via FoxO3a. Exp. Gerontol. 172, 112062. 10.1016/j.exger.2022.112062 36526098

[B249] Zhou H. He Y. Xiong W. Jing S. Duan X. Huang Z. (2023). MSC based gene delivery methods and strategies improve the therapeutic efficacy of neurological diseases. Bioact. Mater 23, 409–437. 10.1016/j.bioactmat.2022.11.007 36474656 PMC9713256

[B250] Zhou T. Yan Y. Zhao C. Xu Y. Wang Q. Xu Na (2019). Resveratrol improves osteogenic differentiation of senescent bone mesenchymal stem cells through inhibiting endogenous reactive oxygen species production via AMPK activation. Redox Rep. 24 (1), 62–69. 10.1080/13510002.2019.1658376 31438780 PMC6748633

[B251] Zhu Y. Tchkonia T. Pirtskhalava T. Gower A. C. Ding H. Giorgadze N. (2015). The Achilles' heel of senescent cells: from transcriptome to senolytic drugs. Aging Cell. 14 (4), 644–658. 10.1111/acel.12344 25754370 PMC4531078

[B252] Zhuang X. Hu X. Zhang S. Li X. Yuan X. Wu Y. (2022). Mesenchymal stem cell-based therapy as a new approach for the treatment of systemic sclerosis. Clin. Rev. Allergy Immunol. 64, 284–320. 10.1007/s12016-021-08892-z 35031958

